# Androgen-Responsive MicroRNAs in Mouse Sertoli Cells

**DOI:** 10.1371/journal.pone.0041146

**Published:** 2012-07-20

**Authors:** Subbarayalu Panneerdoss, Yao-Fu Chang, Kalyan C. Buddavarapu, Hung-I Harry Chen, Gunapala Shetty, Huizhen Wang, Yidong Chen, T. Rajendra Kumar, Manjeet K. Rao

**Affiliations:** 1 Greehey Children's Cancer Research Institute, The University of Texas Health Science Center at San Antonio, San Antonio, Texas, United States of America; 2 Department of Experimental Radiation Oncology, The University of Texas MD Anderson Cancer Center, Houston, Texas, United States of America; 3 Department of Molecular and Integrative Physiology, The University of Kansas Medical Center, Kansas City, Kansas, United States of America; 4 Department of Cellular and Structural Biology, The University of Texas Health Science Center at San Antonio, San Antonio, Texas, United States of America; Clermont Université, France

## Abstract

Although decades of research have established that androgen is essential for spermatogenesis, androgen's mechanism of action remains elusive. This is in part because only a few androgen-responsive genes have been definitively identified in the testis. Here, we propose that microRNAs – small, non-coding RNAs – are one class of androgen-regulated *trans*-acting factors in the testis. Specifically, by using androgen suppression and androgen replacement in mice, we show that androgen regulates the expression of several microRNAs in Sertoli cells. Our results reveal that several of these microRNAs are preferentially expressed in the testis and regulate genes that are highly expressed in Sertoli cells. Because androgen receptor-mediated signaling is essential for the pre- and post-meiotic germ cell development, we propose that androgen controls these events by regulating Sertoli/germ cell-specific gene expression in a microRNA-dependent manner.

## Introduction

Recent studies that conditionally knocked out the androgen receptor (AR) have shown that androgen signaling is necessary for spermatogenesis [1], [2], [3]. However, the molecular mechanisms underlying these events have not been fully elucidated – partly because research has definitively identified only a few androgen-responsive genes in the testis. Among the few genes that we and others have reported are *Rhox5* [4], *Myc* [5], the claudin junction complex protein family (*Cldn1*) [6], *Tgfb1*, *Gsta* [7] and connexin-43/gap junction α1 (*Gja1*) [7]. Most androgen-regulated genes in the testis are probably not direct targets of the AR, but instead respond indirectly to androgen via activation or suppression of transcription by other factors that androgen regulates [8], [9], [10], [11]. These androgen-responsive genes possibly will not have typical androgen response elements but may rather contain regulatory elements in their untranslated region (UTR) to which androgen-responsive trans-acting factors are bound [11], [12].

MicroRNAs (miRNAs) are naturally occurring, short, non-coding RNAs that play important roles in many biological processes, including development and cancer [13], [14]. Increasing number of evidence suggests that miRNAs regulate these events through posttranscriptional control of gene expression, including mRNA degradation, translational repression, DNA methylation and chromatin modification [15], [16]. Because translational repression and chromatin modifications are essential for spermatogenesis [17], [18], and miRNAs are highly expressed in pre- and post-meiotic germ cells [19], [20], miRNAs probably play an important role in postnatal testicular development and function. For example, miR-122a targets transition protein 2 (*Tnp2*), a testis-specific gene involved in chromatin remodeling during mouse spermatogenesis [21]. Furthermore, we and others have reported that germ cell-specific knockout of the miRNA processing enzyme Dicer during embryonic stages [22] and during post-meiotic stages impairs germ cell development and compromises fertility [20]. In addition to germ cell-specific roles, miRNAs play an equally important role in developing Sertoli cells: Sertoli cell-specific Dicer knockout during embryonic stages (using the *Amh-cre* line of mice) resulted in testicular dysgenesis due to alteration in Sertoli cell architecture as early as postnatal day 1 [23]. However, little is known about the role of miRNAs in adult Sertoli cell functions, including androgen-mediated events in the testis.

In this study, we show that testosterone regulates the expression of several miRNAs in adult mouse Sertoli cells. Several of these miRNAs are preferentially expressed in the testis and our results suggest that these miRNAs target genes that are highly expressed in Sertoli and germ cells. Examples include Foxd1, a forkhead/winged-helix transcription factor important in Sertoli cell metabolism [24], and desmocollin-1 (Dsc1), a desmosomal cadherin that plays a crucial role in establishing cell-cell adhesion and desmosome formation in epithelial cells [25]. Because translation repression, a hallmark of miRNA function, is essential for spermatid differentiation [20], our findings suggest that miRNAs may regulate androgen-mediated events either directly, by targeting genes in the AR-responsive Sertoli cells, or indirectly, by regulating germ cell-specific genes.

**Figure 1:**
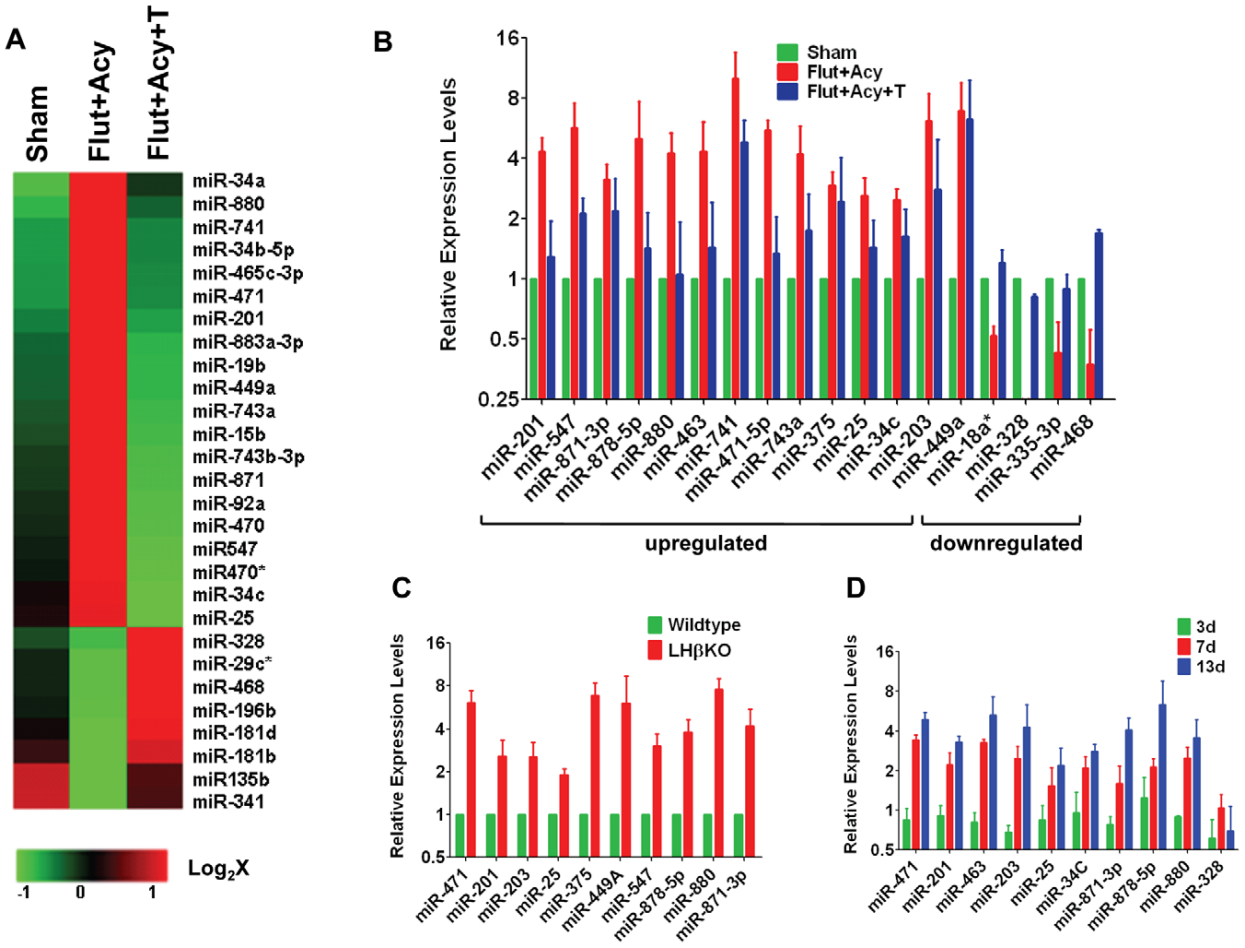
Androgen regulates expression of miRNAs in Sertoli cells. (**A**) Heat map representation of miRNA microarray analyses on total RNA isolated from purified Sertoli cells from control (Sham), flutamide-acyline-treated (Flut+Acy), and flutamide-acyline testosterone-replacement (Flut+Acy+T) mice. Green or red color on the heat map indicates a decrease or increase of miRNA level, respectively, and color intensities correspond to relative signal levels on a logarithmic scale. Sertoli cells were pooled from six mice for each group. (**B**) Real-time RT-PCR analysis (on RNA from purified Sertoli cells) of selected miRNAs using miRNA-specific primers. Shown are selected miRNAs with increased and decreased expression in the absence of androgen and rescued to control levels by testosterone-replacement in the microarray analysis. We pooled Sertoli cells from six mice for each group for each experiment (*n* = 4 for upregulated miRNAs and *n* = 3 for downregulated miRNAs). (**C**) Real-time RT-PCR analysis on RNA from purified Sertoli cells from LHβ KO and sibling control mice (*n* = 3; four mice each) for selected miRNAs. (**D**) Real-time RT-PCR analysis of selected miRNA expression on RNA isolated from Sertoli cells from Sham, Flut+Acy, or Flut+Acy+T mice for indicated days. Results are mean of three different experiments. We pooled Sertoli cells from six mice for each group for each time point. All values for **B**–**D** are normalized against RNU19 levels.

**Figure 2:**
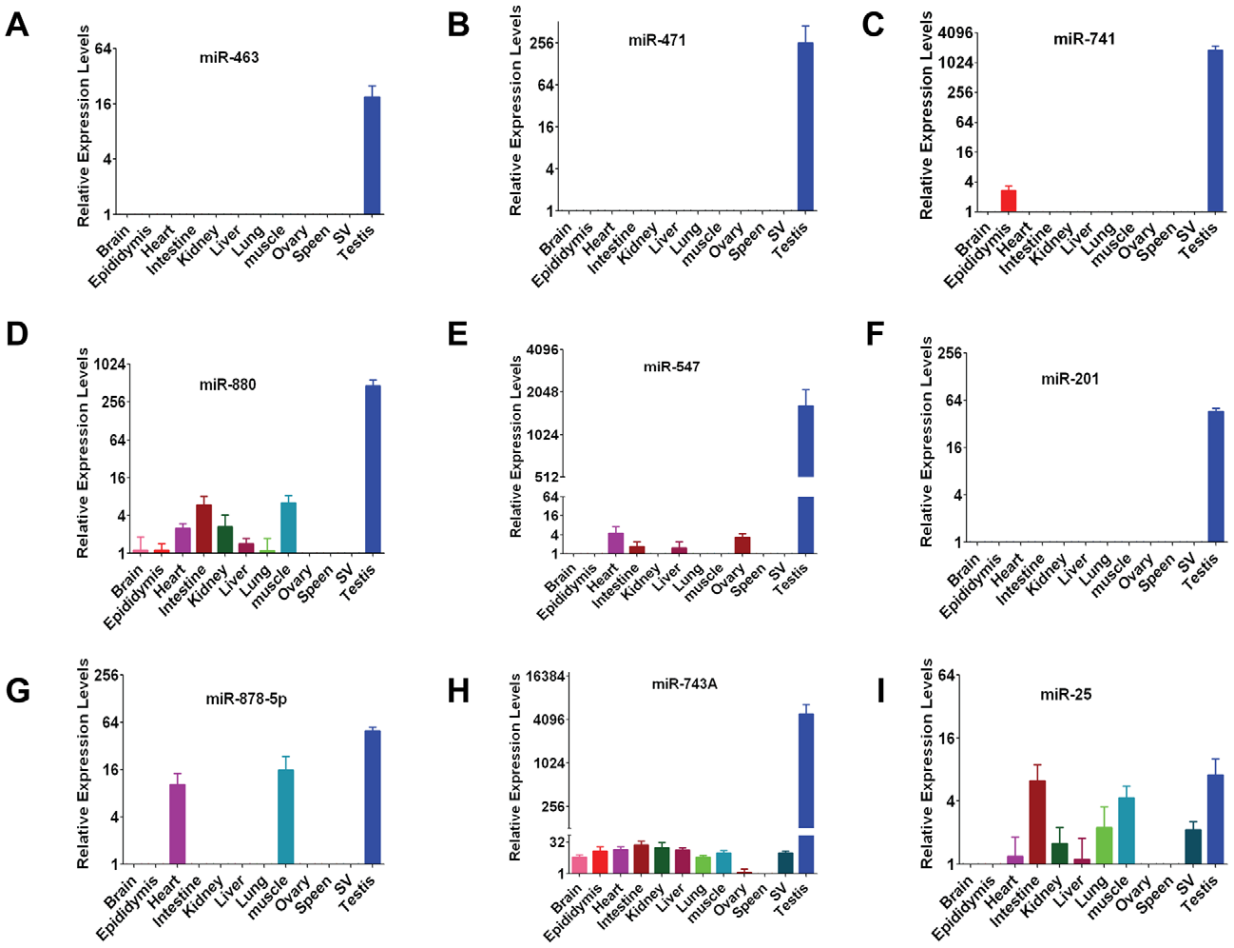
Expression pattern of testosterone-responsive miRNAs. Real-time RT-PCR analysis of selected miRNA expression in total cellular RNA prepared from the adult mouse tissues. All values are normalized against RNU19 levels. Bar graphs represent the mean fold increase ± SEM of miRNA expression over background for at least two RT reactions assayed in duplicate from three separate mice. Several miRNAs negatively regulated by the androgen were specifically expressed in the testis.

**Figure 3:**
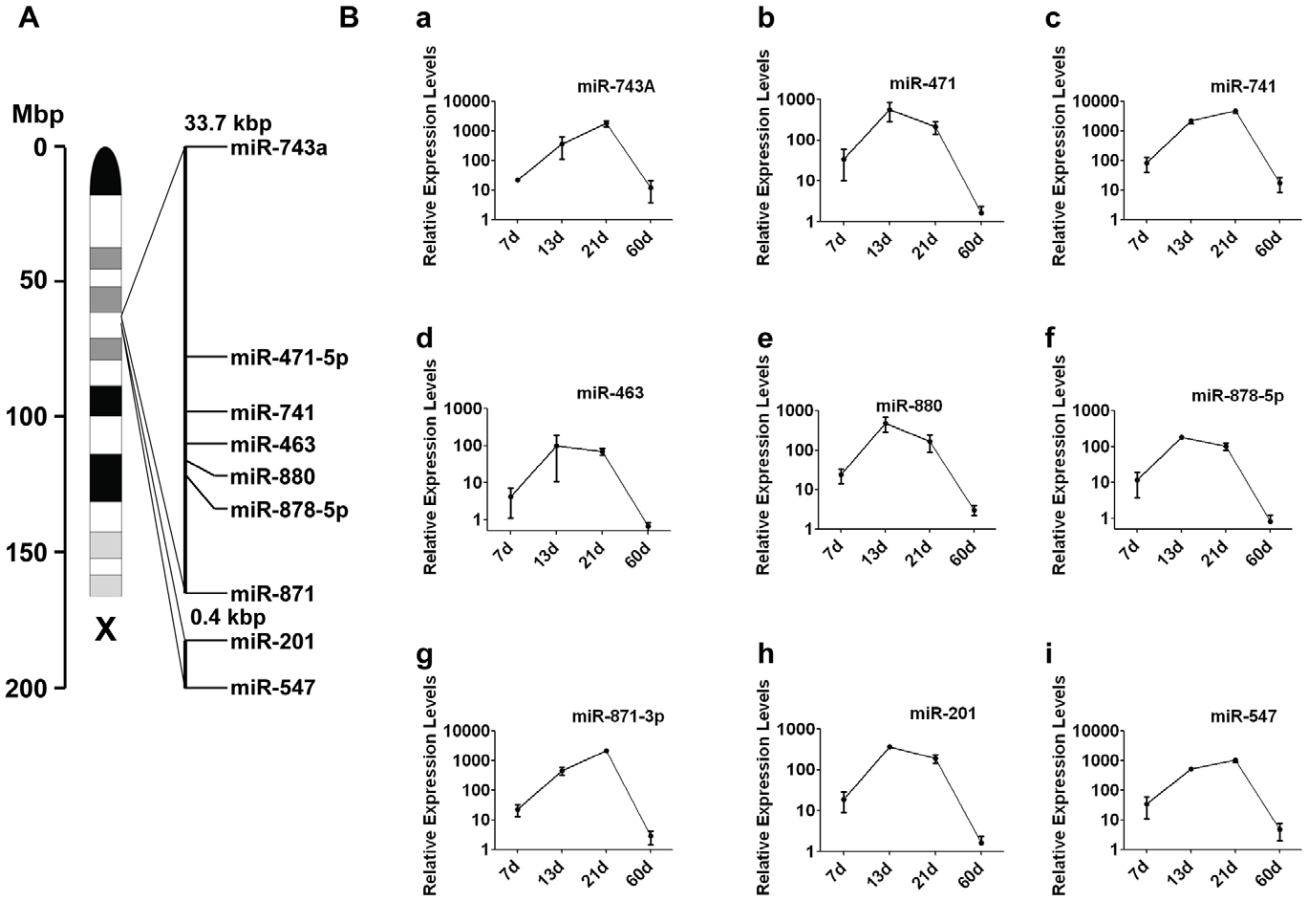
Developmentally regulated expression of testosterone-responsive miRNAs. (**A**) Diagram of the mouse X chromosome showing location of two miRNA clusters. Cluster 1 includes six miRNAs (panels b–g) and cluster 2 includes two miRNAs (panels h and i). (**B**) Real-time RT-PCR analysis of selected miRNA (panels a–h) expression in RNA isolated from purified Sertoli cells from mice of ages shown. Sertoli cells were pooled from seven mice for 7 days (*n = *2 different experiments) and from six mice for each of 13 days (*n = *2 different experiments), 21 days (*n = *3 different experiments), and 60 days (*n = *3 different experiments). All values were normalized against 5S levels. MiRNAs in cluster 2 exhibit quantitative as well as temporal collinear expression pattern.

**Figure 4:**
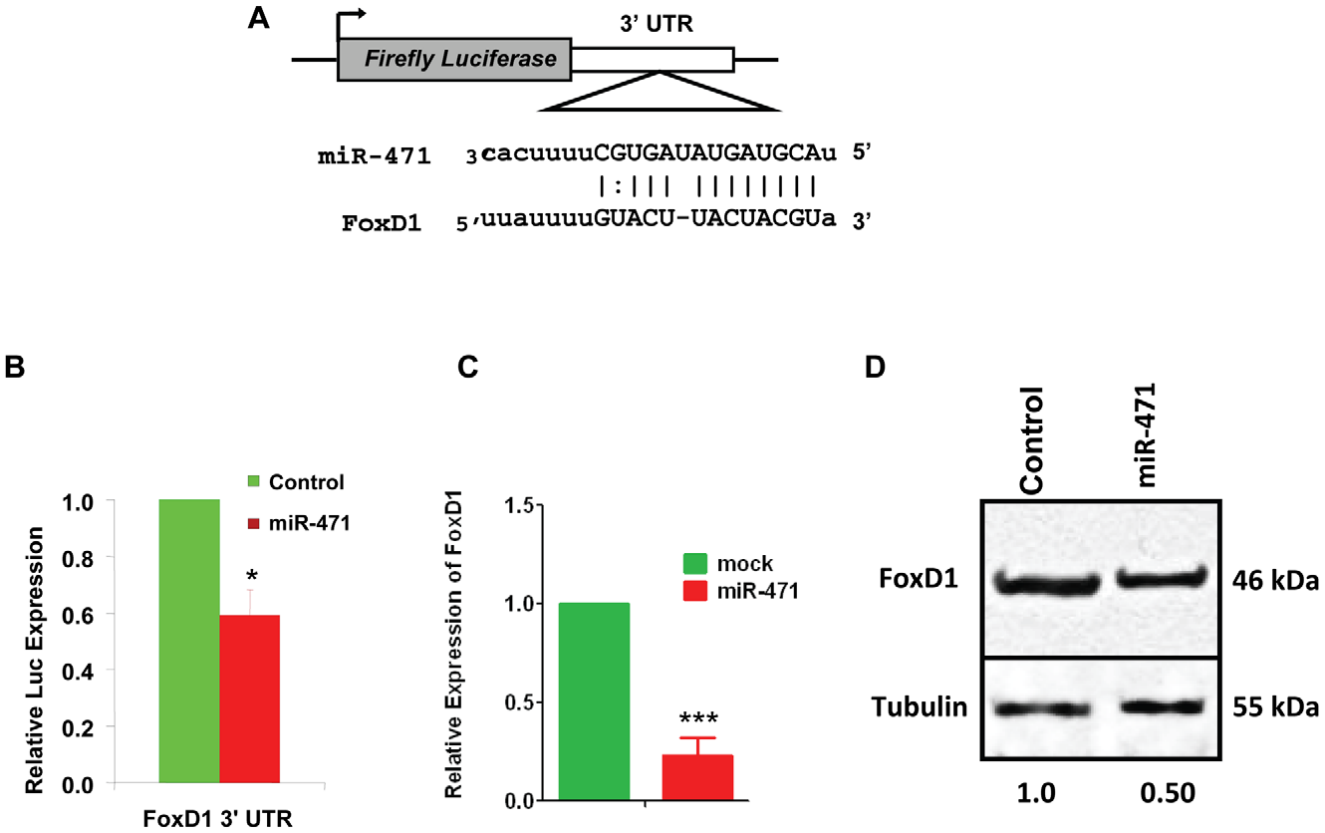
MiR-471 regulates expression of Foxs1 in Sertoli cells. (**A**) Putative miR-471 binding sequence in the *Foxd1* 3′ UTR. (**B**) We co-transfected 15P1 Sertoli cells with *Renilla* luciferase expression construct pRL-CMV and firefly luciferase construct containing pMIR-*Foxd1* 3′ UTR in the absence and presence of miR-471 mimic. We normalized firefly luciferase activity of each sample to *Renilla* luciferase activity. Graphs show mean ± SEM of three independent experiments (performed in duplicate for each experiment). * *p*<0.01; *** *p*<0.001. (**C**) Real-time RT-PCR analysis of miR-471-overexpressing cells by using *Foxd1*-specific primers. (**D**) Western blot analysis of 15P1 cells transfected with miR-471 mimic by using anti-Foxd1 antibody (1∶1000). Tubulin was used as a loading control. Gel photographs represent three independent experiments. Values below the gel were quantified using Image J software (http://rsbweb.nih.gov/ij/). Foxd1 protein level for the control was set to 1.

**Figure 5:**
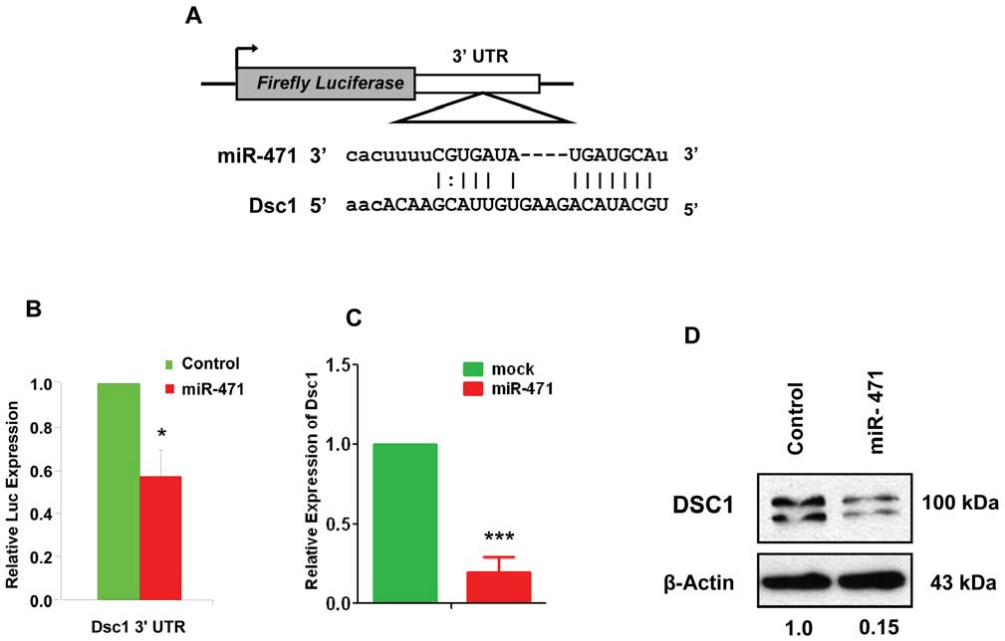
Dsc1 is a bona fide target of miR-471. (**A**) Putative miR-471 binding sequence in the *Dsc1* 3′ UTR. (**B**) We co-transfected 15P1 Sertoli cells with *Renilla* luciferase expression construct pRL-CMV and firefly luciferase construct containing pMIR-*Dsc1* 3′ UTR in the absence and presence of miR-471 mimic. We normalized firefly luciferase activity of each sample to *Renilla* luciferase activity. Graphs show mean ± SEM of three independent experiments (performed in duplicate for each experiment). * *p*<0.01; *** *p*<0.001. (**C**) Real-time RT-PCR analysis of miR-471-overexpressing cells by using *Dsc1*-specific primers. (**D**) Western blot analysis of 15P1 cells transfected with miR-471 mimic by using anti-Dsc1 antibody (1∶250). Actin was used as a loading control. Gel photographs represent three independent experiments. Values below the gel were quantified using the Image J software. Dsc1 protein level for the control was set to 1.

**Figure 6:**
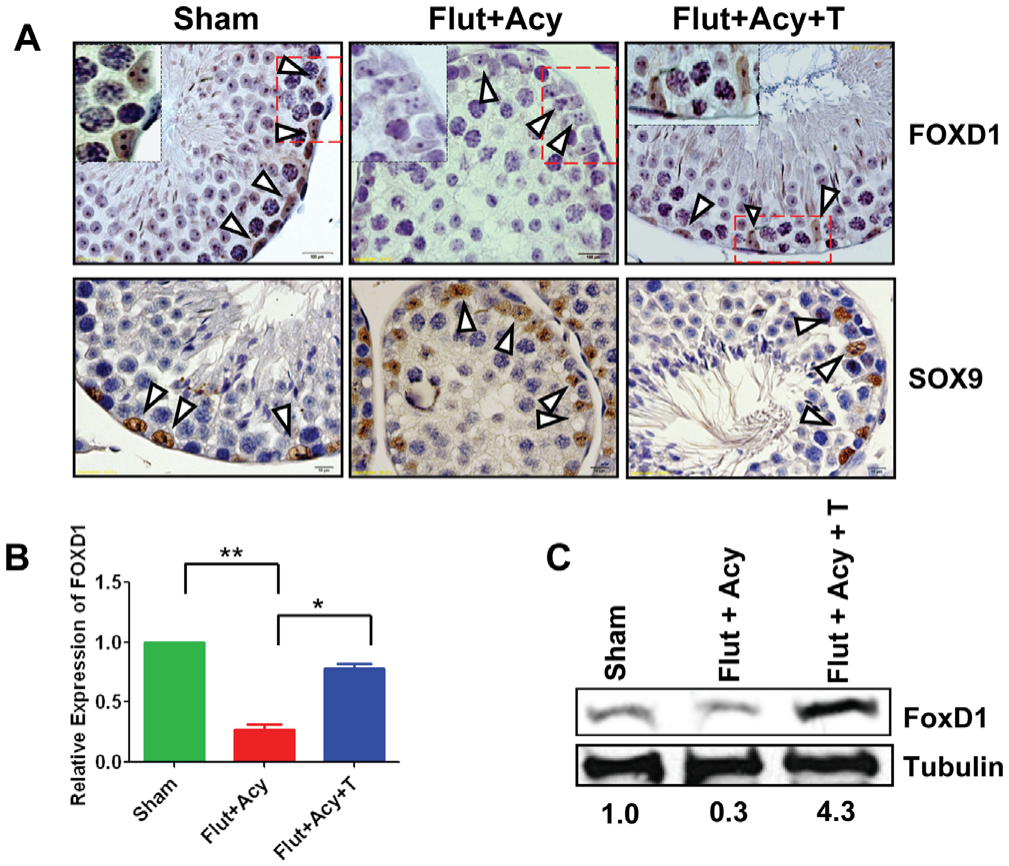
Androgen-dependent expression of FoxD1 in Sertoli cells. (**A**) Immunohistochemical analysis on testis sections from Sham, flutamide-acyline (Flut+Acy) and flutamide-acyline testosterone-replacement (Flut+Acy+T) mice, using antibodies against Foxd1 (1∶50) and Sox9 (1∶500). Insets show Sertoli cell-specific expression of Foxd1 in magnified testicular section from Sham, Flut+Acy, and Flut+Acy+T mice. (**B**) Real-time PCR analysis of *Foxd1* transcript levels in total cellular RNA isolated from the purified Sertoli cells from Sham, Flut+Acy, and Flut+Acy+T mice testes. All values are normalized against RNU19 levels. (**C**) Western blot analysis on total testicular lysates from Sham, Flut+Acy, and Flut+Acy+T mice by using anti-Foxd1 antibody (1∶1000). Tubulin was used as a loading control. Values below the gel were quantified using the Image J software. Foxd1 protein level for the control was set to 1. Arrowheads indicate Sertoli cells. All images were taken at magnification of 600×.

**Figure 7:**
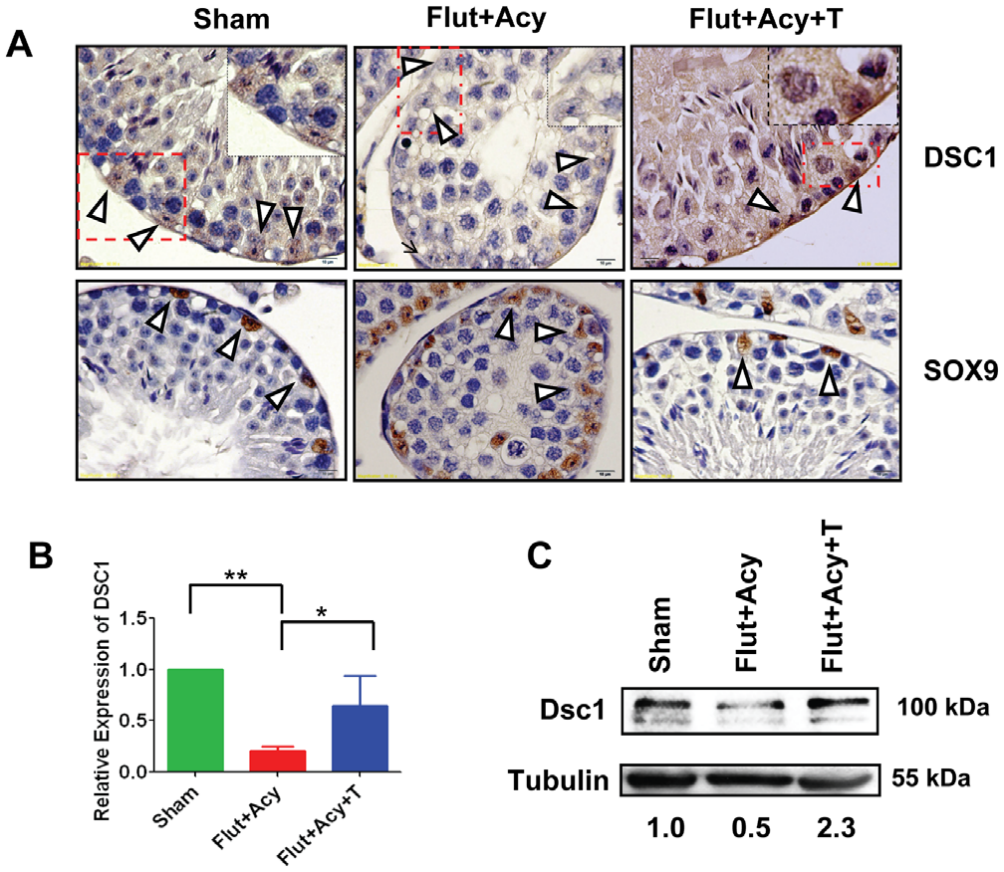
Androgen-dependent expression of Dsc1 in Sertoli cells. (**A**) Immunohistochemical analysis on testis sections from Sham, flutamide-acyline (Flut+Acy) and flutamide-acyline testosterone-replacement (Flut+Acy+T) mice, using antibodies against Dsc1 (1∶50) and Sox9 (1∶500). Insets show Sertoli cell-specific expression of Dsc1 in magnified testicular section from Sham, Flut+Acy, and Flut+Acy+T mice. (**B**) Real-time PCR analysis of *Dsc1* transcript levels in total cellular RNA isolated from the purified Sertoli cells from Sham, Flut+Acy, and Flut+Acy+T mice testes. All values are normalized against RNU19 levels. (**C**) Western blot analysis on total testicular lysates from Sham, Flut+Acy, and Flut+Acy+T mice by using anti-Dsc1 antibody (1∶250). Tubulin was used as a loading control. Values below the gel were quantified using the Image J software. Dsc1 protein level for the control was set to 1. All images were taken at magnification of 600×.

## Results

### Identification of testosterone-responsive miRNAs

To identify testosterone-responsive miRNAs in adult Sertoli cells, we used the antiandrogen drug flutamide and the gonadotropin-releasing hormone (GnRH) antagonist acyline. Previous work has used this androgen-suppression mouse model to validate androgen-regulated genes in the testes of neonatal mice [26]. The flutamide-acyline (Flut+Acy) treatment significantly reduced testicular weight, whereas body weight remained unaltered compared to that of sham-treated (Sham) control mice (Figure S1 and data not shown). The reduced testicular weight was due to loss of germ cells, because flutamide-acyline treatment prevented spermatid development beyond step 8 (Figure S1). Consistent with earlier reports [4], suppression of spermatogenesis in these mice was primarily a consequence of a decrease in intratesticular testosterone levels (Sham = 58.8 ng/g testis weight; Flut+Acy = 2.29 ng/g testis weight). No significant change in the intratesticular levels of testosterone derivative estradiol was observed between sham-treated (40.8±3.0 pg/ml) and flutamide-acyline-treated (42.8±6.0 pg/ml) mice. In mice treated with flutamide-acyline, androgen-supplementation (Flut+Acy+T) rescued intratesticular testosterone levels (Flut+Acy+T = 20.0 ng/g testis weight) and restored spermatogenesis to a level comparable to that of sham-treated control mice. Moreover, the Sertoli cell-specific *Rhox5* homeobox gene, which androgen positively regulates [4], showed significantly decreased expression in mice treated with flutamide-acyline, whereas testosterone-replacement rescued the *Rhox5* levels (Figure S1). To identify androgen-responsive miRNAs, we performed miRNA microarray analysis on RNA isolated from the purified Sertoli cells of sham-treated control mice, mice treated with flutamide-acyline and mice treated with flutamide-acyline and testosterone-replacement (Figure 1A and Table S1). About 38% (218) of the 567 total miRNAs showed detectable levels of expression in normal Sertoli cells. Of the 218 miRNAs expressed in Sertoli cells, we further validated 28 miRNAs showing highly altered levels by real-time RT-PCR analysis (Figure 1B). Compared with sham-treated control mice, most differentially expressed miRNAs were upregulated in the Sertoli cells of mice treated with flutamide-acyline, suggesting that their gene targets would be downregulated without androgen. Testosterone-replacement rescued the expression of several of these miRNAs to levels comparable to those of the sham-treated control group. Interestingly, 18 of 28 highly altered androgen-responsive miRNAs were located on the X chromosome (Table S2).

To further substantiate our findings, we determined the levels of altered miRNAs in LHβ-knockout (LHβ KO) mice. These mice have no detectable levels of intratesticular testosterone compared with wild-type control mice [27]. Consistent with this finding, expression of the androgen-regulated *Rhox5* gene was significantly reduced in LHβ KO mice compared with that of their sibling controls (Figure S1). Real-time RT-PCR analysis on the purified Sertoli cells from LHβ KO mice revealed that testosterone-responsive miRNAs identified in Sertoli cells treated with flutamide-acyline were similarly altered in LHβ KO mice (Figure 1C), further supporting the notion that miRNAs are testosterone-regulated factors in the mouse Sertoli cells. Next, we determined whether miRNAs in Sertoli cells show a time course response to androgen. We compared miRNA levels in mice treated with flutamide-acyline and flutamide-acyline-testosterone-replacement at time points of 3, 7 and 14 days. Most miRNAs showed significantly altered expression only after 14 days of treatment (Figure 1D). This may be due to insufficient reduction in intratesticular testosterone levels observed in day 3 and day 7 treatment groups (data not shown).

### Tissue and developmental specificity of androgen-regulated miRNAs

Besides the testis, several other tissues respond to androgens. Therefore, we asked whether the androgen-responsive miRNAs we identified are expressed only in the testis. By using real-time RT-PCR analysis and primers specific for each miRNA, we determined the levels of 18 highly altered miRNAs in a panel of 12 adult tissues (Figure 2 and Figure S2). All 18 miRNAs were highly expressed in the testis, three of which (miR-463, -471 and -201) were expressed exclusively in the testis (Figure 2). Future studies will determine whether expression (albeit significantly lower than the testis) of some of these miRNAs in other tissues that express androgen receptor is also under the androgen control or whether some tissue-specific factors regulate their expression.

Next, we determined whether these testosterone-responsive miRNAs are developmentally regulated in Sertoli cells. We focused on nine miRNAs (cluster 1: miR-743A, -471-5p, -741, -463, -880, -878-5p and -871; cluster 2: miR-201 and -547) located in two clusters on the X chromosome. Real-time RT-PCR analysis revealed that expression of all these miRNAs in Sertoli cells peaked either at postnatal day 13 or on or after day 21 (Figure 3). These stages are associated with two distinct androgen-dependent steps of spermatogenesis: spermatocytes progress through meiosis I by day 12, and round spermatids begin to differentiate after day 21. MiR-201 and -547 (in cluster 2) displayed a progressive pattern of expression such that the timing and level of their peak expression in Sertoli cells corresponds to their chromosomal position. MiR-201, the most 5′ miRNA with respect to the centromere, is expressed first, and then its expression rapidly ceases. The next miRNA in the cluster, miR-547, is expressed later in development, peaking around day 21. In addition to temporal collinear expression, miR-201 and -547 show quantitative collinearity: miR-201 expression levels are lower than those of miR-547, whose expression peaks when miR-201 expression begins to decline.

### Genes highly expressed in the testis may be putative targets of testosterone-responsive miRNAs

To understand how miRNAs may influence androgen-dependent events during spermatogenesis, we set out to identify genes that testosterone-responsive miRNAs might regulate. Integration of the results from target prediction algorithms (picTar, MirTarget2, Target Scan, miRanda and miTarget) revealed that androgen-responsive miRNAs target several genes involved in testicular physiology (Table S2). Consistent with this finding, pathway analyses of predicted targets showed that androgen-responsive miRNAs target junctional complex, Sertoli cell-germ cell junction signaling and Sertoli cell-Sertoli cell signaling networks (Figure S3). For further analysis, we focused on two target genes of miR-471, forkhead/winged-helix transcription factor Foxd1 and desmosomal cadherin Dsc1, both of which showed highly altered expression in androgen-suppressed and LHβ KO mouse models. Real-time RT-PCR analysis on the purified Sertoli cells showed that while *Foxd1* expression is predominantly in the Sertoli cells, *Dsc1* though highly expressed in the Sertoli cells may also be expressed in the other testicular cell types (Figure S4).

Because miRNAs regulate gene expression by binding to the 3′ UTRs of their target genes, we predicted that miR-471 represses Foxd1 and Dsc1 expression by binding to their 3′ UTRs. Bioinformatics analyses revealed that both *Foxd1* and *Dsc1* 3′ UTRs contained one putative binding site for miR-471 (Figure 4A and Figure 5A). To determine whether miR-471 indeed binds to *Foxd1* and *Dsc1* 3′ UTRs at their putative binding sites, we transfected Sertoli cells with a pMIR-REPORT vector construct containing either the *Foxd1* or *Dsc1* 3′ UTRs and then measured luciferase activity. As shown in Figure 4B and Figure 5B, luciferase activity was significantly repressed in pMIR-*Foxd1* and -*Dsc1* 3′ UTR transfected 15P1 Sertoli cells when compared with the null pMIR construct. Finally, we assessed the effect of miR-471 on Foxd1 and Dsc1 transcript and protein levels by performing real-time RT-PCR and western blot analysis in 15P1 Sertoli cells and HeLa cells overexpressing a miR-471 oligonucleotide (miR-471 mimic). Overexpression of miR-471 mimic significantly reduced Foxd1 and Dsc1 RNA (Figure 4C and Figure 5C) and protein (Figure 4D, Figure 5D and Figure S4) levels. Collectively, our results confirmed that miR-471 regulates FoxD1 and Dsc1 expression at both the RNA and protein levels by binding to their 3′ UTRs.

### Androgen-dependent regulation of Foxd1 and Dsc1 in Sertoli cells

Next we sought to determine whether testosterone indeed controls expression of Foxd1 and Dsc1 in Sertoli cell in vivo. We performed immunohistochemical analysis on testicular sections from sham-treated control, flutamide-acyline-treated and flutamide-treated testosterone-replacement mice. In comparison with control mice, flutamide treatment significantly lowered Foxd1 and Dsc1, whereas testosterone-replacement rescued their expressions in the Sertoli cells (Figure 6A and Figure 7A). In contrast to Foxd1 and Dsc1, expression of Sertoli cell-specific protein Sox9, which is not regulated by the androgen in the adult testis, showed no change in flutamide-acyline-treated or testosterone-supplemented groups when compared with the sham-treated control group. Supporting this, real-time RT-PCR analysis on the purified Sertoli cells from control, flutamide-acyline-treated and testosterone-supplementation mice testes showed significantly lower *Foxd1* and *Dsc1* levels in the flutamide-acyline-treated mice when compared to control mice, while testosterone-supplementation rescued their transcripts levels comparable to the control mice (Figure 6B and Figure 7B). Western blot analysis on testicular lysates from control, flutamide-acyline-treated and flutamide-treated androgen-replacement mice further substantiated these findings (Figure 6C and Figure 7C). Taken together, these findings suggest that testosterone-responsive miR-471 regulate expression of Foxd1 and Dsc1, which are expressed in a testosterone-dependent manner in the mouse Sertoli cells. Future studies manipulating the levels of miR-471 in the Sertoli cells in vivo will further validate these findings.

## Discussion

Although androgen activation of adult Sertoli cells is essential for germ cell development and differentiation [1], [2], the underlying molecular mechanism remains largely unknown. Here, we identify miRNAs as one group of testosterone-dependent trans-acting factors in postnatal Sertoli cells that may play a crucial role in androgen-mediated events during spermatogenesis by targeting Sertoli cell/germ cell-specific genes. Our results reveal that several of these testosterone-responsive miRNAs are expressed in a developmentally-dependent manner in the mouse Sertoli cells. Many miRNAs (including miR-471, -470, -463, -465, -743a/b, -883, -880 -201 and -547) are located in clusters on the X chromosome, suggesting that they may play a coordinated role in androgen-dependent spermatogenic events. Furthermore, our pathway analysis indicates that these miRNAs may target genes or factors involved in cellular signaling and junction restructuring, two androgen-dependent processes crucial for germ cell development. Our results showing increased levels of several miRNAs in the absence of testosterone propose that testosterone-mediated inhibition of miRNAs in Sertoli cells may facilitates the expression of genes essential for spermatogenesis.

Our results show that expression of Dsc1, a calcium-dependent glycoprotein expressed in desmosomes at the epithelial cellular junctions [25], is regulated by testosterone-responsive miR-471 in the Sertoli cell lines. Although a direct role for Dsc1 in the testis has not been identified, mice lacking Dsc1 exhibit epidermal fragility due to defects in cell adhesion and barrier functions [25]. Furthermore, other desmosomal proteins, such as Dsc2 and desmogleins, play an important role in junctional restructuring by regulating tight junction protein zonula occludens-1 (ZO-1) at the blood-testis-barrier (BTB) [28]. These findings suggest that Dsc1 may also play a similar role in Sertoli cell-Sertoli cell adhesion at the BTB. Because rapid recycling of signaling proteins is vital for extensive junctional restructuring at the BTB to facilitate germ cell translocation and maintain BTB integrity [29], testosterone-responsive miRNAs probably coordinate this process by tightly controlling the expression of junctional proteins, including Dsc1.

In addition to BTB, the continued expression of androgen-responsive miRNAs in postnatal Sertoli cells suggests that they may play an equally essential role in other androgen-dependent processes, including post-meiotic germ cell development, Sertoli cell-spermatid adhesion and sperm release. Consistent with this assertion, we demonstrate that miR-471 targets forkhead/winged-helix transcription factor Foxd1, which induces cAMP-dependent expression of protein kinase A (PKA) [24]. PKA activation phosphorylates and later activates many cellular proteins, including CREB, a Sertoli cell-specific transcription factor crucial in pos-tmeiotic germ cell development and maturation [9], [30]. PKA-dependent phosphorylation of CREB also transcriptionally activates several Sertoli cell genes within hours of testosterone stimulation [9]. Because this rapid, testosterone-dependent transcription occurs through CREB phosphorylation, inhibiting androgen-responsive miRNAs even slightly will probably profoundly affect Sertoli cell function by altering the levels of transcription factors, such as Foxd1, that mediate PKA activation and CREB phosphorylation.

Recently, a study in rats reported that androgen regulates several miRNAs [31]. However, the miRNAs that our study identified do not correlate with those in that study, for at least two reasons. First, the rat study used androgen-supplementation to elicit a change in miRNA expression, whereas we used androgen-suppression. Second, differences in miRNAs and their expression pattern between mice and rats may account for the disparity in the two reports.

In conclusion, our study establishes the importance of miRNAs, a new class of regulatory molecules in testicular physiology. To our knowledge, our study is the first to address the mechanism by which miRNAs, the androgen-responsive trans-acting factors in Sertoli cells, may regulate androgen-dependent events essential for male fertility. Because miRNAs can regulate many factors in single or multiple regulatory pathways, tightly controlled expression of androgen-responsive miRNAs is vital for spermatogenesis. Future studies aimed at addressing the regulation and precise function of the androgen-responsive miRNAs could provide deeper understanding of how androgen regulates Sertoli cell physiology and various events in spermatogenesis.

## Materials and Methods

### Animals

All animal experiments were performed in accordance with the National Institutes of Health Guide for the Care and Use of Laboratory Animals. Approval of animal use for this study was granted by The Institutional Animal Care and Use Committee of The University of Texas Health Science Center at San Antonio (Animal Welfare Assurance #A3345-01; Protocol #10072X) and The University of Kansas Medical Center (ACUP #2010-1930). We maintained B6129 male mice aged 5–7 weeks (Taconic, Hudson, NY) in a fixed 12 h light-dark cycle with free access to food and water. We generated and genotyped LHβ KO mice as previously described [27]. To identify androgen-responsive miRNAs in Sertoli cells, we divided mice into three treatment groups:

1. Sham (0.2 cm Silastic tube implanted subcutaneously [s.c.] and saline injected intraperitoneally [i.p.])
2. Antiandrogen flutamide (Sigma, St. Louis, MO; administered through a 0.2 cm Silastic tube implanted s.c.) and anti-GnRH acyline (20 mg/kg body weight injected i.p.)
3. Androgen-replacement (flutamide and testosterone [Sigma; administered through a 0.2 cm Silastic tube implanted s.c.] and acyline [20 mg/kg body weight injected i.p.])

### Sertoli cell purification and RNA isolation

We euthanized mice by asphyxiation with CO2, harvested their testes, and isolated Sertoli cells as previously described [32], [33]. We extracted RNA from purified Sertoli cells (pooled from five to seven mice) by using the miRNeasy Mini Kit (Qiagen, Valencia, CA). We converted 1 µg of total RNA into cDNA by using the miScript Reverse Transcription Kit (Qiagen) and analyzed with real-time RT-PCR (7900HT Fast Real-time PCR System; Life Technologies, Grand Island, NY; cycling conditions: 15 s at 95°C, 30 s at 55°C, 30 s at 70°C, 40 cycles) using the miScript SYBR Green PCR Kit (Qiagen) and miRNA-specific forward primers. We normalized miRNA expression levels to RNU19/5S and gene expression to 18S. Table S3 lists primer sequences for both miRNAs and genes.

### Microarray and real-time RT-PCR validation

We hybridized purified Sertoli cell RNA to the Agilent 4×44 k Whole Mouse Genome Microarray (Agilent Technologies, Santa Clara, CA) according to manufacturer's protocol and scanned with the Agilent G2505B scanner. We extracted relative miRNA expression ratios with Agilent's Feature Extraction software. We normalized quantiles with the MATLAB Bioinformatics Toolbox (R2009a; Mathworks, Natick, MA). Selected signature miRNA sets were obtained by calculating normalized log_2_ ratio using ((log_2_ data)-μ)/s algorithm, where μ is the average log_2_ intensity of each miRNA among three samples and s is the standard deviation of each miRNA among three samples. Only miRNAs with normalized ratios ≥2 fold (log_2_ 1) were selected. Microarray results were validated using real-time RT-PCR analysis as explained above. Differential expression of miRNAs between groups was tested for significance using a single-factor analysis of variance (ANOVA) with the Tukey multiple comparison test. *p*<0.05 was taken as a level of significance. The differential miRNA expression dataset has been deposited in the GEO/NCBI database (GSE37679).

### Immunohistochemistry and western blot analysis

We carried out immunohistochemical analyses on 4% paraformaldehyde (PFA)-fixed and paraffin-embedded testicular sections as previously described [4]. We performed western blot analysis on testis extracts from sham control (Sham), flutamide-acyline (Flut+Acy), and androgen-replacement (Flut+Acy+T) groups or cell extracts from miR-471 mimic-transfected 15P1 Sertoli cells and HeLa cells as described previously [34]. We purchased antibodies to actin (A5316; Sigma), tubulin (556321; BD Biosciences, San Jose, CA), Foxd1 (SC-133586; Santa Cruz Biotechnology, Santa Cruz, CA), Sox9 (Santa Cruz Biotechnology) and Dsc1 (SC-18115; Santa Cruz Biotechnology and AP8666b; Abgent, San Diego, CA).

### Hormone measurements

We measured intratesticular testosterone by radioimmunoassay, using the Coat-A-Count Kit (PITKITT 5; Siemens, Tarrytown, NY), as described previously [35]. Intratesticular estradiol was measured using ELISA at the Ligand Assay and Analysis Core, University of Virginia School of Medicine, Charlottesville, VA.

### Plasmids

The 3′ UTR segments of the *Foxd1* and *Dsc1* genes were PCR amplified and subcloned into the pMIR-REPORT vector (Life Technologies) at the HindIII and SpeI restriction sites to generate the pMIR-*Foxd1* and pMIR-*Dsc1* 3′ UTR-containing plasmids.

### Luciferase assay

We co-transfected HeLa cells with 500 ng of luciferase-3′ UTR constructs and 5 ng of pRL-CMV (Promega, Madison, WI) using Lipofectamine 2000 (Life Technologies). Twenty-four hours after transfection, we transfected cells with miRNA mimics (Life Technologies) at a final concentration of 100 nM using Lipofectamine 2000. We harvested cells after an additional 48 h according to manufacturer's protocol from the Dual Luciferase Reporter Assay System (Promega) and read luciferase activity by using the Glomax 20/20 Luminometer (Promega).

### Statistical analysis

All values and error bars in graphs are means ± SEM; respective *n*-values are indicated in figure legends; *p*-values were determined by two-tailed Student's *t*-tests and ANOVA with the Tukey multiple comparison test.

## Supporting Information

Figure S1 **Anti-androgen treatment suppresses spermatogenesis.** (**A**) Photomicrograph showing reduced testicular size in flutamide-acyline-treated (Flut+Acy) mice and rescued testis size in flutamide-acyline testosterone-supplementation (Flut+Acy+T) mice when compared to sham-treated (Sham) control mice. (**B**) Bar graph showing testis weight in Sham, Flut+Acy and Flut+Acy+T mice. (**C**) Histological analysis on testicular sections showing normal spermatogenesis in Sham, suppression of spermatogenesis beyond step 8 in Flut+Acy and resumption of spermatogenesis in Flut+Acy+T mice. (**D**) Real-time RT-PCR analysis using *Rhox5*-specific primers on purified Sertoli cells show significantly decreased levels of *Rhox5* in Flut+Acy mice and rescued *Rhox5* levels in Flut+Acy+T mice when compared to Sham control. Sertoli cells were pooled from 6 mice for each group for each experiment (*n* = 3 different experiments). (**E**) Real-time RT-PCR analysis using *Rhox5*-specific primers on purified Sertoli cells show significantly decreased levels of *Rhox5* in LHβ KO mice when compared to sibling control.
(TIF)

Figure S2 **Expression pattern of androgen-responsive miRNAs.** Real-time RT-PCR analysis of selected miRNA expression in total cellular RNA prepared from the adult mice tissues shown. All values are normalized against RNU19 levels. Bar graphs represent the mean fold increase ± SEM of miRNA expression over background for at least two RT reactions assayed in duplicate from three separate mice.
(TIF)

Figure S3 **Pathway analyses of predicted androgen-responsive miRNA targets.** Biological pathway analyses of predicted target genes of differentially expressed miRNAs (>2-fold threshold obtained from microarray) using Ingenuity Systems IPA software (Redwood City, CA). Target genes associated with selected biological functions and canonical pathways in the Ingenuity Knowledge Base were considered for the analysis. Fischer's exact test was used to calculate a *p*-value determining the probability that each biological function and canonical pathway assigned to the candidate miRNA target genes is due to chance alone. Biological functions and canonical pathways with *p*<0.05 were considered significant.
(TIF)

Figure S4 **Foxd1 and Dsc1 are expressed in the Sertoli cells and targeted by miR-471.** (**A**) Real-time RT-PCR analyses to determine Foxd1 and Dsc1 transcripts levels in purified Sertoli cells relative to total testis. The values shown are representative of three independent experiments. (**B**) Western blot analysis of HeLa cells transfected with miR-471 mimic by using anti-Foxd1 (1∶1000) or anti-Dsc1 antibody (1∶250). Actin was used as a loading control. Gel photographs represent three independent experiments.
(TIF)

Table S1 Partial list of differentially expressed miRNAs. Fold difference (shown as log_2_) for miRNAs altered in Flut+Acy and Flut+Acy+T groups are calculated as log_2_ (Flut+Acy)/log_2_ (Sham) and log_2_ (Flut+Acy+T)/log_2_ (Flut+Acy), respectively. Data showing the complete list of androgen-responsive miRNAs are deposited to the GEO database (GSE37679).
(DOC)

Table S2 List of predicted genes targeted by androgen-responsive miRNAs. MiRNAs identified from the microarray analysis (Table S1).
(DOC)

Table S3 Primers used in this study.
(DOC)
