# Supplementary figures and images for: Androgen-Responsive MicroRNAs in Mouse Sertoli Cells
Source: PLoS One. 2012 Jul 20;7(7):e41146. doi: 10.1371/journal.pone.0041146 (PMC3401116; doi:10.1371/journal.pone.0041146)

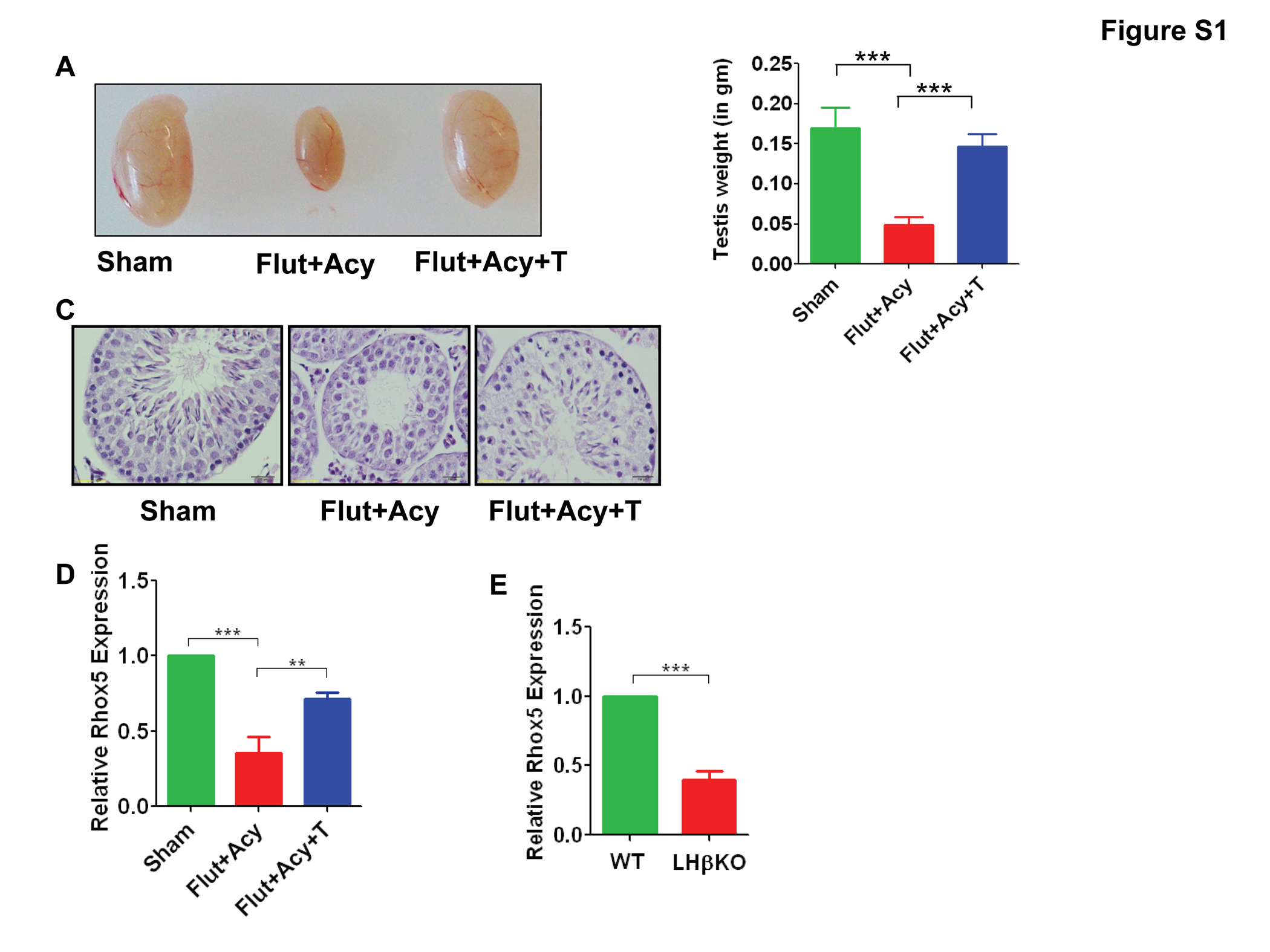

Supplement: Figure S1 — Anti-androgen treatment suppresses spermatogenesis. (A) Photomicrograph showing reduced testicular size in flutamide-acyline-treated (Flut+Acy) mice and rescued testis size in flutamide-acyline testosterone-supplementation (Flut+Acy+T) mice when compared to sham-treated (Sham) control mice. (B) Bar graph showing testis weight in Sham, Flut+Acy and Flut+Acy+T mice. (C) Histological analysis on testicular sections showing normal spermatogenesis in Sham, suppression of spermatogenesis beyond step 8 in Flut+Acy and resumption of spermatogenesis in Flut+Acy+T mice. (D) Real-time RT-PCR analysis using Rhox5-specific primers on purified Sertoli cells show significantly decreased levels of Rhox5 in Flut+Acy mice and rescued Rhox5 levels in Flut+Acy+T mice when compared to Sham control. Sertoli cells were pooled from 6 mice for each group for each experiment (n = 3 different experiments). (E) Real-time RT-PCR analysis using Rhox5-specific primers on purified Sertoli cells show significantly decreased levels of Rhox5 in LHβ KO mice when compared to sibling control. (TIF) [file pone.0041146.s001.tif]

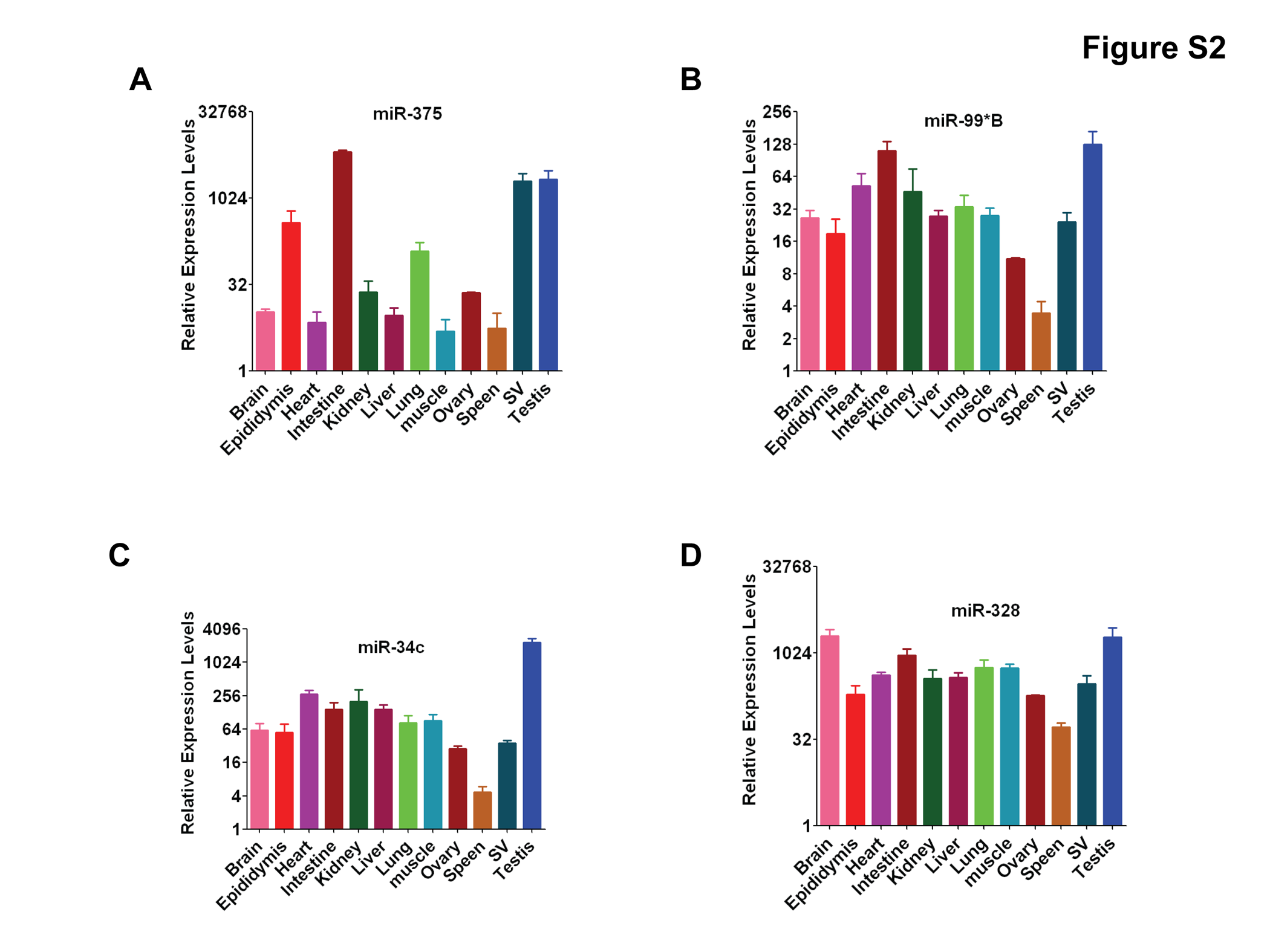

Supplement: Figure S2 — Expression pattern of androgen-responsive miRNAs. Real-time RT-PCR analysis of selected miRNA expression in total cellular RNA prepared from the adult mice tissues shown. All values are normalized against RNU19 levels. Bar graphs represent the mean fold increase ± SEM of miRNA expression over background for at least two RT reactions assayed in duplicate from three separate mice. (TIF) [file pone.0041146.s002.tif]

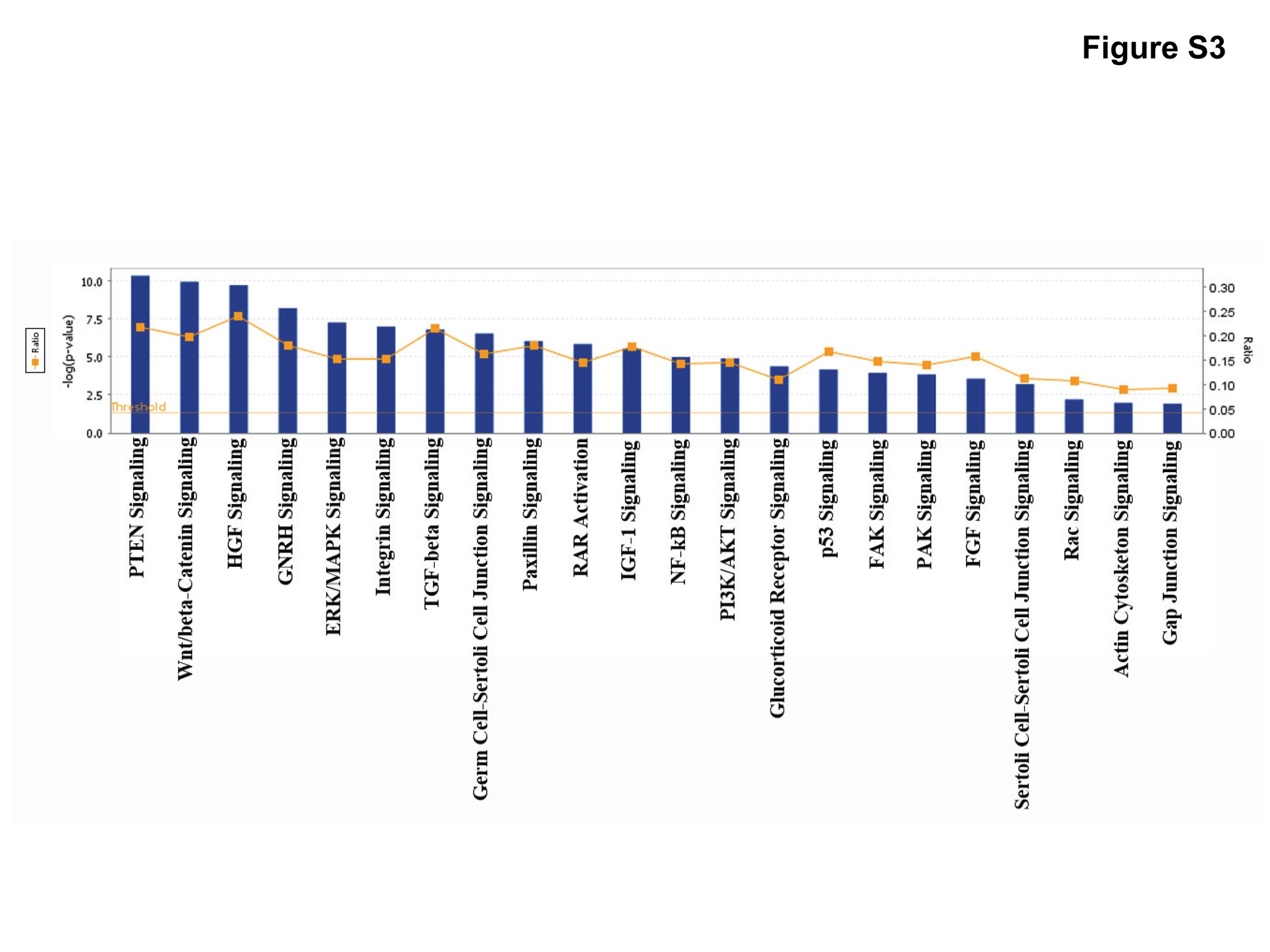

Supplement: Figure S3 — Pathway analyses of predicted androgen-responsive miRNA targets. Biological pathway analyses of predicted target genes of differentially expressed miRNAs (>2-fold threshold obtained from microarray) using Ingenuity Systems IPA software (Redwood City, CA). Target genes associated with selected biological functions and canonical pathways in the Ingenuity Knowledge Base were considered for the analysis. Fischer's exact test was used to calculate a p-value determining the probability that each biological function and canonical pathway assigned to the candidate miRNA target genes is due to chance alone. Biological functions and canonical pathways with p<0.05 were considered significant. (TIF) [file pone.0041146.s003.tif]

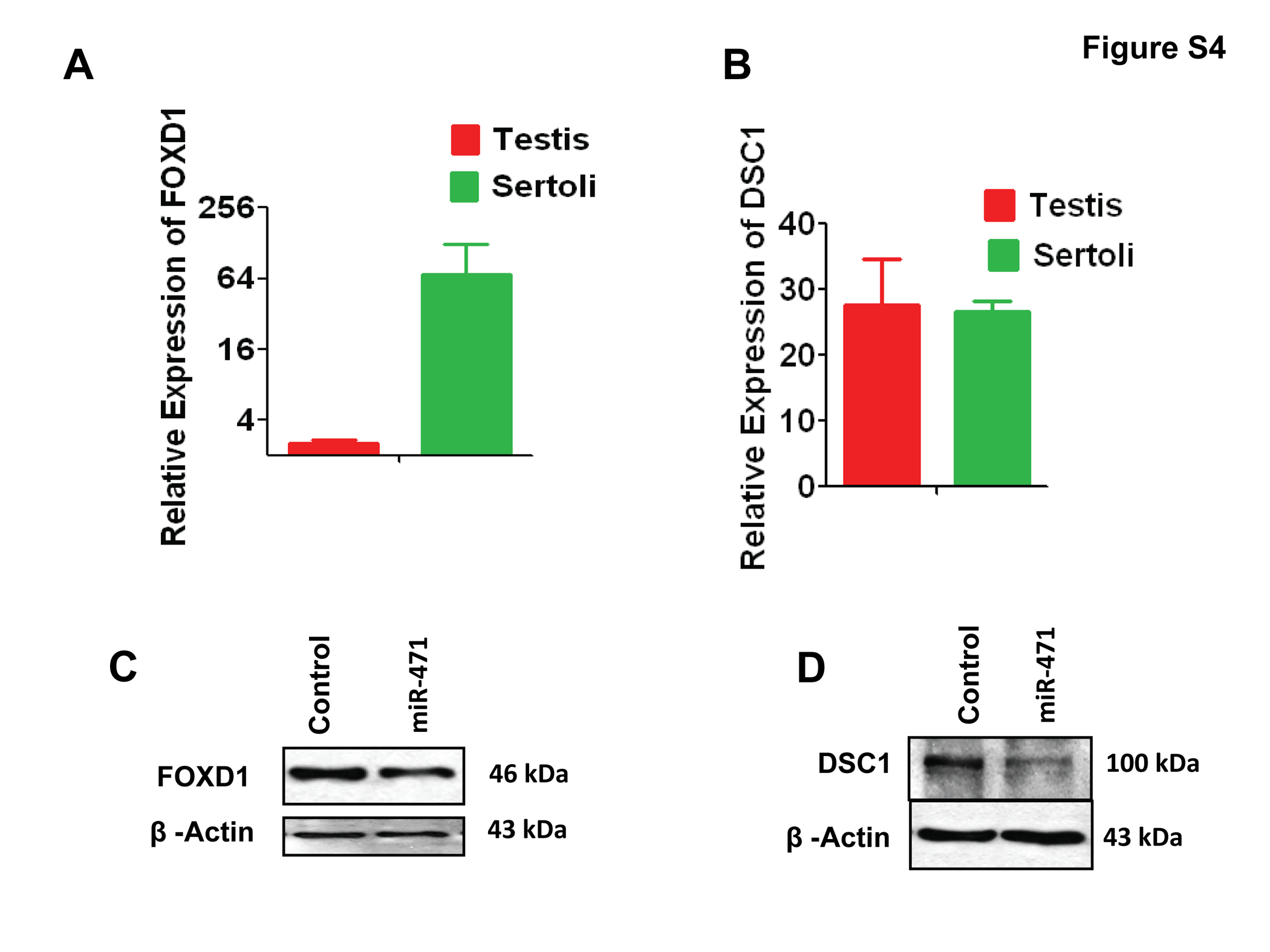

Supplement: Figure S4 — Foxd1 and Dsc1 are expressed in the Sertoli cells and targeted by miR-471. (A) Real-time RT-PCR analyses to determine Foxd1 and Dsc1 transcripts levels in purified Sertoli cells relative to total testis. The values shown are representative of three independent experiments. (B) Western blot analysis of HeLa cells transfected with miR-471 mimic by using anti-Foxd1 (1∶1000) or anti-Dsc1 antibody (1∶250). Actin was used as a loading control. Gel photographs represent three independent experiments. (TIF) [file pone.0041146.s004.tif]
