# Supplementary material for: Androgen-Responsive MicroRNAs in Mouse Sertoli Cells
Source: PLoS One. 2012 Jul 20;7(7):e41146. doi: 10.1371/journal.pone.0041146 (PMC3401116; doi:10.1371/journal.pone.0041146)
Supplement: Table S1 — Partial list of differentially expressed miRNAs. Fold difference (shown as log2) for miRNAs altered in Flut+Acy and Flut+Acy+T groups are calculated as log2 (Flut+Acy)/log2 (Sham) and log2 (Flut+Acy+T)/log2 (Flut+Acy), respectively. Data showing the complete list of androgen-responsive miRNAs are deposited to the GEO database (GSE37679). (DOC) [file pone.0041146.s005.doc]

Table S1. Partial list of differentially expressed miRNAs.

| **MiRNA** | **Fold Difference Flut+Acy / Sham (log_2_)** | **Fold Difference Flut+Acy+T / Flut+Acy (log_2_)** | **Chromosome** |
| --- | --- | --- | --- |
| mmu-miR-129-3p | 2.405810397 | 0.33152209 | 6 |
| mmu-miR-15b | 2.581853379 | 0.28798371 | 3 |
| mmu-miR-19a | 3.06421229 | 0.243262633 | 14 |
| mmu-miR-19b | 2.818034753 | 0.280157575 | 14 & X |
| mmu-miR-201 | 4.149378841 | 0.218643234 | X |
| mmu-miR-547 | 3.099642108 | 0.20853129 | X |
| mmu-miR-25 | 2.044262761 | 0.23834558 | 5 |
| mmu-miR-34a | 7.172064167 | 0.266912935 | 4 |
| mmu-miR-34b-5p | 4.551991536 | 0.238642559 | 9 |
| mmu-miR-34c | 3.153054317 | 0.136994787 | 9 |
| mmu-miR-34c* | 2.110672812 | 0.398659591 | 9 |
| mmu-miR-375 | 2.257480599 | 0.37063825 | 1 |
| mmu-miR-449a | 3.566539107 | 0.216227209 | 13 |
| mmu-miR-463 | 2.753186329 | 0.369941838 | X |
| mmu-miR-465c-3p | 5.471269651 | 0.192409136 | X |
| mmu-miR-466e-3p | 2.185106881 | 0.402373494 | 2 |
| mmu-miR-470 | 3.425233552 | 0.195242735 | X |
| mmu-miR-470* | 3.425233552 | 0.181947205 | X |
| mmu-miR-471 | 4.38985355 | 0.233840911 | X |
| mmu-miR-741 | 5.524972388 | 0.197156675 | X |
| mmu-miR-743a | 5.612930093 | 0.120015053 | X |
| mmu-miR-743b-3p | 6.119425181 | 0.096724632 | X |
| mmu-miR-871 | 3.69827187 | 0.202651876 | X |
| mmu-miR-878-5p | 2.577233096 | 0.354512798 | X |
| mmu-miR-880 | 5.599541969 | 0.237623836 | X |
| mmu-miR-883a-3p | 4.185752707 | 0.191423163 | X |
| mmu-miR-883a-5p | 2.996875649 | 0.232472528 | X |
| mmu-miR-883b-3p | 2.059459321 | 0.394933507 | X |

Fold difference (shown as log_2_) for miRNAs altered in Flut+Acy and Flut+Acy+T groups are calculated as log_2_ (Flut+Acy) / log_2_ (Sham) and log_2_ (Flut+Acy+T) / log_2_ (Flut+Acy), respectively. Data showing the complete list of androgen-responsive miRNAs are deposited to the GEO database (GSE37679).
