# Supplementary material for: Androgen-Responsive MicroRNAs in Mouse Sertoli Cells
Source: PLoS One. 2012 Jul 20;7(7):e41146. doi: 10.1371/journal.pone.0041146 (PMC3401116; doi:10.1371/journal.pone.0041146)
Supplement: Table S2 — List of predicted genes targeted by androgen-responsive miRNAs. MiRNAs identified from the microarray analysis (Table S1). (DOC) [file pone.0041146.s006.doc]

**Table S2. List of predicted genes targeted by androgen-responsive miRNAs.**

Gene Symbol miRNA

Cep97 mmu-miR-471

Creg1 mmu-miR-471

Cxcl16 mmu-miR-471

Dsc1 mmu-miR-471

Dsc2 mmu-miR-471

Fam73a mmu-miR-471

Fign mmu-miR-471

Foxd1 mmu-miR-471

Il33 mmu-miR-471

Nfat5 mmu-miR-471

Ptgr2 mmu-miR-471

Rdh20 mmu-miR-471

Slc9a3r1 mmu-miR-471

Smarcc2 mmu-miR-471

Smyd3 mmu-miR-471

Snf1lk mmu-miR-471

Ssbp2 mmu-miR-471

Sv2b mmu-miR-471

Tef mmu-miR-471

Tmem161b mmu-miR-471

Xkrx mmu-miR-471

Aebp2 mmu-miR-463

Barhl1 mmu-miR-463

Camk1d mmu-miR-463

Ccdc71 mmu-miR-463

Dtx1 mmu-miR-463

Eif4g2 mmu-miR-463

Eml4 mmu-miR-463

Foxp2 mmu-miR-463

Gabrb3 mmu-miR-463

Iqck mmu-miR-463

Lmo4 mmu-miR-463

Lypla1 mmu-miR-463

Man1a mmu-miR-463

Nedd4l mmu-miR-463

Pcgf3 mmu-miR-463

Prpf4 mmu-miR-463

Ralbp1 mmu-miR-463

Rbpms mmu-miR-463

Rod1 mmu-miR-463

Smg1 mmu-miR-463

Timp3 mmu-miR-463

Tmem49 mmu-miR-463

Ubfd1 mmu-miR-463

Gene Symbol miRNA

Aff4 mmu-miR-878-5p

Ankrd11 mmu-miR-878-5p

Apc mmu-miR-878-5p

Arrdc3 mmu-miR-878-5p

Baz1a mmu-miR-878-5p

Bcl2l11 mmu-miR-878-5p

Btf3l4 mmu-miR-878-5p

Cnih4 mmu-miR-878-5p

Csnk1a1 mmu-miR-878-5p

Ddx3x mmu-miR-878-5p

Eif4e mmu-miR-878-5p

Epc2 mmu-miR-878-5p

Eps8 mmu-miR-878-5p

Gdap1 mmu-miR-878-5p

Gfod1 mmu-miR-878-5p

Igfbp3 mmu-miR-878-5p

Igfbpl1 mmu-miR-878-5p

Ipo8 mmu-miR-878-5p

Itgb3bp mmu-miR-878-5p

Mbnl1 mmu-miR-878-5p

Mid2 mmu-miR-878-5p

Mns1 mmu-miR-878-5p

Msrb3 mmu-miR-878-5p

Mum1l1 mmu-miR-878-5p

Nr6a1 mmu-miR-878-5p

Ogt mmu-miR-878-5p

Pax6 mmu-miR-878-5p

Prss35 mmu-miR-878-5p

Ptpn9 mmu-miR-878-5p

Rad23b mmu-miR-878-5p

Rc3h2 mmu-miR-878-5p

Rnf24 mmu-miR-878-5p

Runx1t1 mmu-miR-878-5p

Sephs1 mmu-miR-878-5p

Slc16a10 mmu-miR-878-5p

Slc37a2 mmu-miR-878-5p

Slc39a10 mmu-miR-878-5p

Slc40a1 mmu-miR-878-5p

Syt1 mmu-miR-878-5p

Tmed10 mmu-miR-878-5p

Tnrc6b mmu-miR-878-5p

Wdr19 mmu-miR-878-5p

Wtap mmu-miR-878-5p

Ythdf3 mmu-miR-878-5p

Gene Symbol miRNA

Ahctf1 mmu-miR-880

Ankrd11 mmu-miR-880

Anks1b mmu-miR-880

Arhgef9 mmu-miR-880

Atrn mmu-miR-880

Braf mmu-miR-880

Cbx4 mmu-miR-880

Ccnyl1 mmu-miR-880

Cggbp1 mmu-miR-880

Chst12 mmu-miR-880

Clec7a mmu-miR-880

Commd7 mmu-miR-880

Corin mmu-miR-880

Dennd5b mmu-miR-880

Dgkk mmu-miR-880

Dixdc1 mmu-miR-880

Ell mmu-miR-880

Evi5 mmu-miR-880

Fam188b mmu-miR-880

Fam199x mmu-miR-880

Fbxo11 mmu-miR-880

Fos mmu-miR-880

Grm5 mmu-miR-880

Hapln1 mmu-miR-880

Icmt mmu-miR-880

Igfbp5 mmu-miR-880

Inoc1 mmu-miR-880

Jarid2 mmu-miR-880

Luc7l2 mmu-miR-880

Map2k1ip1 mmu-miR-880

Mapk9 mmu-miR-880

Mesdc1 mmu-miR-880

Mllt11 mmu-miR-880

Mtap2 mmu-miR-880

Patl1 mmu-miR-880

Pcbp2 mmu-miR-880

Per1 mmu-miR-880

Phf21b mmu-miR-880

Plagl2 mmu-miR-880

Ppp3r1 mmu-miR-880

Rarb mmu-miR-880

Slc23a2 mmu-miR-880

Smad6 mmu-miR-880

Stx3 mmu-miR-880

Syncrip mmu-miR-880

Gene Symbol miRNA

Tardbp mmu-miR-880

Tcf12 mmu-miR-880

Timp3 mmu-miR-880

Tnrc18 mmu-miR-880

Trpv4 mmu-miR-880

Tsc22d2 mmu-miR-880

Usp54 mmu-miR-880

Usp9x mmu-miR-880

Wtap mmu-miR-880

Zfp148 mmu-miR-880

Zfp182 mmu-miR-880

Zfp46 mmu-miR-880

Zfp827 mmu-miR-880

Arhgap26 mmu-miR-741

Arhgef12 mmu-miR-741

Arih1 mmu-miR-741

Arpc5 mmu-miR-741

Barhl1 mmu-miR-741

Btrc mmu-miR-741

C2cd2l mmu-miR-741

Cacna1e mmu-miR-741

Ccdc38 mmu-miR-741

Cntd1 mmu-miR-741

Cops2 mmu-miR-741

Cpne3 mmu-miR-741

Derl2 mmu-miR-741

Dnajc14 mmu-miR-741

Dynll2 mmu-miR-741

Efnb3 mmu-miR-741

Elp4 mmu-miR-741

Epc1 mmu-miR-741

Esrrg mmu-miR-741

Fam107b mmu-miR-741

Fam49a mmu-miR-741

Fam78b mmu-miR-741

Frmpd4 mmu-miR-741

Gpr173 mmu-miR-741

Gria2 mmu-miR-741

Hlcs mmu-miR-741

Kif5a mmu-miR-741

Lrp4 mmu-miR-741

Maoa mmu-miR-741

Mcl1 mmu-miR-741

Mfap3 mmu-miR-741

Mixl1 mmu-miR-741

Gene Symbol miRNA

Ncan mmu-miR-741

Nptx1 mmu-miR-741

Numa1 mmu-miR-741

Onecut2 mmu-miR-741

Otud7b mmu-miR-741

Pcmtd1 mmu-miR-741

Phldb1 mmu-miR-741

Pknox1 mmu-miR-741

Ppp3r1 mmu-miR-741

Pscd1 mmu-miR-741

Rhoq mmu-miR-741

Rnf114 mmu-miR-741

Rrp8 mmu-miR-741

Slc9a5 mmu-miR-741

Snx30 mmu-miR-741

Sobp mmu-miR-741

Tbl2 mmu-miR-741

Tbpl1 mmu-miR-741

Tcfap2b mmu-miR-741

Tnrc4 mmu-miR-741

Traf3 mmu-miR-741

Trp63 mmu-miR-741

Tspan9 mmu-miR-741

Txn2 mmu-miR-741

Txndc13 mmu-miR-741

Zbtb4 mmu-miR-741

Zfp780b mmu-miR-741

Adarb1 mmu-miR-201

Adcy1 mmu-miR-201

Ankrd40 mmu-miR-201

Arhgef12 mmu-miR-201

Bach2 mmu-miR-201

Bard1 mmu-miR-201

Cblb mmu-miR-201

Col4a5 mmu-miR-201

Creb1 mmu-miR-201

Egr1 mmu-miR-201

Ept1 mmu-miR-201

Esyt2 mmu-miR-201

Fam78b mmu-miR-201

Fbxl20 mmu-miR-201

Fbxo8 mmu-miR-201

Flrt3 mmu-miR-201

Fosl2 mmu-miR-201

Gabrb3 mmu-miR-201

Gene Symbol miRNA

H3f3a mmu-miR-201

Hoxa1 mmu-miR-201

Hs3st3a1 mmu-miR-201

Ikzf3 mmu-miR-201

Immt mmu-miR-201

Kcnc1 mmu-miR-201

Kif5a mmu-miR-201

Kras mmu-miR-201

Maf1 mmu-miR-201

Med13 mmu-miR-201

Mms19 mmu-miR-201

Mycbp2 mmu-miR-201

Ncam1 mmu-miR-201

Nr2f2 mmu-miR-201

Nudt18 mmu-miR-201

Otud4 mmu-miR-201

Pappa mmu-miR-201

Ppp2r5e mmu-miR-201

Prkd3 mmu-miR-201

Rap2b mmu-miR-201

Rapgef6 mmu-miR-201

Refbp2 mmu-miR-201

Ret mmu-miR-201

Rev3l mmu-miR-201

Rnf144a mmu-miR-201

Rpa1 mmu-miR-201

Scai mmu-miR-201

Sema6a mmu-miR-201

Sox11 mmu-miR-201

Sox9 mmu-miR-201

Srpk2 mmu-miR-201

St8sia2 mmu-miR-201

Stk16 mmu-miR-201

Tcf4 mmu-miR-201

Tsc22d2 mmu-miR-201

Tshz3 mmu-miR-201

Ubxd2 mmu-miR-201

Wnt2b mmu-miR-201

Zbtb7c mmu-miR-201

Arhgef40 mmu-miR-547

Cul4a mmu-miR-547

Diap1 mmu-miR-547

Dixdc1 mmu-miR-547

Dnajc14 mmu-miR-547

Dnmt3b mmu-miR-547

Gene Symbol miRNA

Dr1 mmu-miR-547

Dse mmu-miR-547

Dync1li1 mmu-miR-547

Dyrk1a mmu-miR-547

Dyrk1b mmu-miR-547

Efna3 mmu-miR-547

Elk1 mmu-miR-547

Enah mmu-miR-547

Enpep mmu-miR-547

Esr1 mmu-miR-547

Esyt2 mmu-miR-547

Fbxo11 mmu-miR-547

Fndc3a mmu-miR-547

Foxn2 mmu-miR-547

Fyttd1 mmu-miR-547

Gad2 mmu-miR-547

Galnt3 mmu-miR-547

Gjd2 mmu-miR-547

Gls mmu-miR-547

Gnai3 mmu-miR-547

Gnas mmu-miR-547

Gpr45 mmu-miR-547

Gzf1 mmu-miR-547

Hapln1 mmu-miR-547

Hnrnpab mmu-miR-547

Icmt mmu-miR-547

Igf1 mmu-miR-547

Ikzf2 mmu-miR-547

Ing3 mmu-miR-547

Inpp5a mmu-miR-547

Ipo4 mmu-miR-547

Kif26a mmu-miR-547

Kitl mmu-miR-547

Klf4 mmu-miR-547

Mafb mmu-miR-547

Maml1 mmu-miR-547

Map3k2 mmu-miR-547

Map3k3 mmu-miR-547

Map4k4 mmu-miR-547

Mbd2 mmu-miR-547

Mbnl2 mmu-miR-547

Mdga1 mmu-miR-547

Mdga2 mmu-miR-547

Mesdc1 mmu-miR-547

Mga mmu-miR-547

Gene Symbol miRNA

Mkln1 mmu-miR-547

Mpp3 mmu-miR-547

Mtap1b mmu-miR-547

Mtmr2 mmu-miR-547

Myo1c mmu-miR-547

Narg1 mmu-miR-547

Ndst3 mmu-miR-547

Nfib mmu-miR-547

Nptx1 mmu-miR-547

Onecut2 mmu-miR-547

Onecut3 mmu-miR-547

Orc4l mmu-miR-547

Pafah1b1 mmu-miR-547

Paip1 mmu-miR-547

Pbx1 mmu-miR-547

Pde8b mmu-miR-547

Pdgfc mmu-miR-547

Pdgfrb mmu-miR-547

Phactr4 mmu-miR-547

Pik3r1 mmu-miR-547

Pik3r3 mmu-miR-547

Pitpnm2 mmu-miR-547

Plagl1 mmu-miR-547

Ppara mmu-miR-547

Ppp2ca mmu-miR-547

Prdm16 mmu-miR-547

Prdm6 mmu-miR-547

Prkcb1 mmu-miR-547

Prps2 mmu-miR-547

Prtg mmu-miR-547

Prune mmu-miR-547

Ptchd1 mmu-miR-547

Ptgfrn mmu-miR-547

Ptpdc1 mmu-miR-547

Ptprg mmu-miR-547

Ptprj mmu-miR-547

Pygo2 mmu-miR-547

Qk mmu-miR-547

Rap1b mmu-miR-547

Rap2b mmu-miR-547

Rbpj mmu-miR-547

Rcor1 mmu-miR-547

Reep4 mmu-miR-547

Rell1 mmu-miR-547

Rhot1 mmu-miR-547

Gene Symbol miRNA

Rnf144a mmu-miR-547

Rnf19a mmu-miR-547

Rnft1 mmu-miR-547

Rpia mmu-miR-547

Rrbp1 mmu-miR-547

Rufy2 mmu-miR-547

Runx1t1 mmu-miR-547

Scn8a mmu-miR-547

Setd8 mmu-miR-547

Sh3bgrl2 mmu-miR-547

Shc1 mmu-miR-547

Sin3a mmu-miR-547

Slc10a3 mmu-miR-547

Slc17a6 mmu-miR-547

Slc19a2 mmu-miR-547

Slc20a2 mmu-miR-547

Slc22a23 mmu-miR-547

Slc25a26 mmu-miR-547

Slc2a3 mmu-miR-547

Slc35e1 mmu-miR-547

Slc38a2 mmu-miR-547

Slc39a14 mmu-miR-547

Slc44a5 mmu-miR-547

Slc5a3 mmu-miR-547

Slc7a14 mmu-miR-547

Snap91 mmu-miR-547

Sntb2 mmu-miR-547

Son mmu-miR-547

Spag9 mmu-miR-547

Specc1l mmu-miR-547

Spg21 mmu-miR-547

Spock1 mmu-miR-547

St8sia4 mmu-miR-547

Stt3a mmu-miR-547

Stx16 mmu-miR-547

Syap1 mmu-miR-547

Syncrip mmu-miR-547

Tanc2 mmu-miR-547

Tbpl1 mmu-miR-547

Tgfbr1 mmu-miR-547

Tln2 mmu-miR-547

Tmcc3 mmu-miR-547

Tmed2 mmu-miR-547

Tmed8 mmu-miR-547

Tmem109 mmu-miR-547

Gene Symbol miRNA

Tmem150 mmu-miR-547

Tmem16a mmu-miR-547

Tmem179b mmu-miR-547

Tmod2 mmu-miR-547

Tnc mmu-miR-547

Tnpo1 mmu-miR-547

Trim2 mmu-miR-547

Trim33 mmu-miR-547

Trmt6 mmu-miR-547

Trove2 mmu-miR-547

Tsc22d2 mmu-miR-547

Tulp4 mmu-miR-547

Ubap2 mmu-miR-547

Ube2g1 mmu-miR-547

Ube2q1 mmu-miR-547

Ube4b mmu-miR-547

Ubp1 mmu-miR-547

Usp14 mmu-miR-547

Vav3 mmu-miR-547

Wdr44 mmu-miR-547

Ywhag mmu-miR-547

Zbtb37 mmu-miR-547

Zbtb4 mmu-miR-547

Zbtb44 mmu-miR-547

Zfhx3 mmu-miR-547

Zfhx4 mmu-miR-547

Zfp148 mmu-miR-547

Zfp217 mmu-miR-547

Zfp462 mmu-miR-547

Zfp710 mmu-miR-547

Zfp827 mmu-miR-547

Zic1 mmu-miR-547

Zmat3 mmu-miR-547

Apln mmu-miR-743a

Arf6 mmu-miR-743a

Arid2 mmu-miR-743a

Arid4a mmu-miR-743a

Arl15 mmu-miR-743a

Asxl2 mmu-miR-743a

Cadm4 mmu-miR-743a

Camk2b mmu-miR-743a

Camkv mmu-miR-743a

Ccdc88a mmu-miR-743a

Cd200r3 mmu-miR-743a

Cdk2ap2 mmu-miR-743a

Gene Symbol miRNA

Fgf4 mmu-miR-743a

Fgfr1 mmu-miR-743a

Foxp2 mmu-miR-743a

Fzd7 mmu-miR-743a

Gpr56 mmu-miR-743a

Gprc5b mmu-miR-743a

Grk5 mmu-miR-743a

Hoxb2 mmu-miR-743a

Igfbp4 mmu-miR-743a

Igfbp5 mmu-miR-743a

Il13ra1 mmu-miR-743a

Ilf3 mmu-miR-743a

Impad1 mmu-miR-743a

Ing2 mmu-miR-743a

Inhbb mmu-miR-743a

Ipmk mmu-miR-743a

Iqsec2 mmu-miR-743a

Itga9 mmu-miR-743a

Jarid2 mmu-miR-743a

Map3k1 mmu-miR-743a

Mapkapk2 mmu-miR-743a

Rab10 mmu-miR-743a

Rab5b mmu-miR-743a

Rap1b mmu-miR-743a

Rap2c mmu-miR-743a

Rapgef2 mmu-miR-743a

Tgfbr2 mmu-miR-743a

Tgfbr3 mmu-miR-743a

Tiprl mmu-miR-743a

Wnt7a mmu-miR-743a

Bmi1 mmu-miR-203

Bmper mmu-miR-203

Bptf mmu-miR-203

Ccnd1 mmu-miR-203

Ccnd2 mmu-miR-203

Ccng1 mmu-miR-203

Ccrn4l mmu-miR-203

Cdc14b mmu-miR-203

Cdc2l5 mmu-miR-203

Cdh10 mmu-miR-203

Centg2 mmu-miR-203

Chd2 mmu-miR-203

Chd9 mmu-miR-203

Dcc mmu-miR-203

Dcp2 mmu-miR-203

Gene Symbol miRNA

Dcx mmu-miR-203

Ddx6 mmu-miR-203

Derl2 mmu-miR-203

Dgcr2 mmu-miR-203

Eya4 mmu-miR-203

Fgf16 mmu-miR-203

Foxk1 mmu-miR-203

Foxk2 mmu-miR-203

Foxp2 mmu-miR-203

Fyco1 mmu-miR-203

Fyn mmu-miR-203

Gpr109a mmu-miR-203

Gpr180 mmu-miR-203

Gpr81 mmu-miR-203

Grhl3 mmu-miR-203

Kctd9 mmu-miR-203

Khdrbs1 mmu-miR-203

Kif1b mmu-miR-203

Kif2a mmu-miR-203

Kpnb1 mmu-miR-203

Map3k1 mmu-miR-203

Map3k13 mmu-miR-203

Mapk10 mmu-miR-203

Mapre1 mmu-miR-203

Mecp2 mmu-miR-203

Ncam1 mmu-miR-203

Nfil3 mmu-miR-203

Pten mmu-miR-203

Ptp4a1 mmu-miR-203

Ptprg mmu-miR-203

Pcdh10 mmu-miR-203

Pcdh19 mmu-miR-203

Pclo mmu-miR-203

Rap1a mmu-miR-203

Rap2a mmu-miR-203

Rassf10 mmu-miR-203

Smad3 mmu-miR-203

Src mmu-miR-203

Stx16 mmu-miR-203

Ubr1 mmu-miR-203

Ubxd6 mmu-miR-203

Ugcg mmu-miR-203

Upf2 mmu-miR-203

Zcchc8 mmu-miR-203

Zdhhc20 mmu-miR-203

Gene Symbol miRNA

Zfp148 mmu-miR-203

Arhgap1 mmu-miR-34c

Arhgap26 mmu-miR-34c

Arid4a mmu-miR-34c

Arl15 mmu-miR-34c

Asb1 mmu-miR-34c

Ascl1 mmu-miR-34c

Atg4b mmu-miR-34c

Bcl2 mmu-miR-34c

Cacna1e mmu-miR-34c

Cacnb3 mmu-miR-34c

Cacng2 mmu-miR-34c

Camsap1 mmu-miR-34c

Camta1 mmu-miR-34c

Cant1 mmu-miR-34c

Car7 mmu-miR-34c

Casp2 mmu-miR-34c

Cbfa2t3 mmu-miR-34c

Ccnd1 mmu-miR-34c

Ccne2 mmu-miR-34c

Cdc25a mmu-miR-34c

Cdh4 mmu-miR-34c

Chl1 mmu-miR-34c

Clcn3 mmu-miR-34c

Clock mmu-miR-34c

Cnot4 mmu-miR-34c

Cntn2 mmu-miR-34c

Cntnap1 mmu-miR-34c

Ctnnd1 mmu-miR-34c

Dcp1a mmu-miR-34c

Dcx mmu-miR-34c

Ddx17 mmu-miR-34c

Fbxo30 mmu-miR-34c

Fgd6 mmu-miR-34c

Fndc3b mmu-miR-34c

Fndc8 mmu-miR-34c

Foxg1 mmu-miR-34c

Foxj2 mmu-miR-34c

Foxn2 mmu-miR-34c

Gas1 mmu-miR-34c

Igfbp3 mmu-miR-34c

Kcnd3 mmu-miR-34c

Kcne1l mmu-miR-34c

Kcnk3 mmu-miR-34c

Map2k1 mmu-miR-34c

Gene Symbol miRNA

Met mmu-miR-34c

Metap1 mmu-miR-34c

Notch1 mmu-miR-34c

Notch2 mmu-miR-34c

Pdgfra mmu-miR-34c

Pou3f3 mmu-miR-34c

Ppargc1b mmu-miR-34c

Ppp1r10 mmu-miR-34c

Ppp1r11 mmu-miR-34c

Rab43 mmu-miR-34c

Ralgds mmu-miR-34c

Ralgps2 mmu-miR-34c

Scn2b mmu-miR-34c

Sdhc mmu-miR-34c

Spry3 mmu-miR-34c

Srpr mmu-miR-34c

Stac2 mmu-miR-34c

Stat6 mmu-miR-34c

Stk38l mmu-miR-34c

Strn3 mmu-miR-34c

Stx17 mmu-miR-34c

Tnrc18 mmu-miR-34c

Tnrc4 mmu-miR-34c

Wnt1 mmu-miR-34c

Camsap1 mmu-miR-19b

Caprin1 mmu-miR-19b

Cbx6 mmu-miR-19b

Cbx7 mmu-miR-19b

Bcl3 mmu-miR-19b

Card10 mmu-miR-19b

Cc2d1a mmu-miR-19b

Ccdc126 mmu-miR-19b

Ccdc142 mmu-miR-19b

Ccdc88a mmu-miR-19b

Ccm2 mmu-miR-19b

Ccnd1 mmu-miR-19b

Ccnd2 mmu-miR-19b

Ccnl1 mmu-miR-19b

Ccrn4l mmu-miR-19b

Clock mmu-miR-19b

Cnot4 mmu-miR-19b

Cpeb3 mmu-miR-19b

Crebl2 mmu-miR-19b

Cyld mmu-miR-19b

Dcp2 mmu-miR-19b

Gene Symbol miRNA

Dcun1d2 mmu-miR-19b

Ddef2 mmu-miR-19b

Ddx3x mmu-miR-19b

Dgcr8 mmu-miR-19b

Dicer1 mmu-miR-19b

Dlc1 mmu-miR-19b

Dlx1 mmu-miR-19b

Dlx3 mmu-miR-19b

Elk3 mmu-miR-19b

Eea1 mmu-miR-19b

Efnb2 mmu-miR-19b

Epc1 mmu-miR-19b

Epc2 mmu-miR-19b

Epgn mmu-miR-19b

Ephb3 mmu-miR-19b

Epn2 mmu-miR-19b

Ereg mmu-miR-19b

Flnc mmu-miR-19b

Fndc3a mmu-miR-19b

Id2 mmu-miR-19b

Igf2r mmu-miR-19b

Igfbp3 mmu-miR-19b

Igsf3 mmu-miR-19b

Il6st mmu-miR-19b

Impdh1 mmu-miR-19b

Ing5 mmu-miR-19b

Inhbb mmu-miR-19b

Inoc1 mmu-miR-19b

Itch mmu-miR-19b

Itga6 mmu-miR-19b

Jarid2 mmu-miR-19b

Kcna4 mmu-miR-19b

Kcnb2 mmu-miR-19b

Kcnj2 mmu-miR-19b

Kif1b mmu-miR-19b

Kif3a mmu-miR-19b

Kit mmu-miR-19b

Map3k12 mmu-miR-19b

Map3k14 mmu-miR-19b

Map3k2 mmu-miR-19b

Map3k7ip3 mmu-miR-19b

Mapk1 mmu-miR-19b

Mapk10 mmu-miR-19b

Mapk14 mmu-miR-19b

Mapk6 mmu-miR-19b

Gene Symbol miRNA

Mef2a mmu-miR-19b

Mycn mmu-miR-19b

Pcdh10 mmu-miR-19b

Pcdha1 mmu-miR-19b

Pcdha10 mmu-miR-19b

Pcdha11 mmu-miR-19b

Pcdha12 mmu-miR-19b

Pcdha2 mmu-miR-19b

Pcdha3 mmu-miR-19b

Pcdha4 mmu-miR-19b

Pcdha5 mmu-miR-19b

Pcdha6 mmu-miR-19b

Pcdha7 mmu-miR-19b

Pcdha8 mmu-miR-19b

Pcdha9 mmu-miR-19b

Pcdhac1 mmu-miR-19b

Pcdhac2 mmu-miR-19b

Ppp1r12a mmu-miR-19b

Ppp1r9a mmu-miR-19b

Ppp2r5e mmu-miR-19b

Pptc7 mmu-miR-19b

Pten mmu-miR-19b

Rab21 mmu-miR-19b

Rab2b mmu-miR-19b

Rab33b mmu-miR-19b

Rab34 mmu-miR-19b

Rab5b mmu-miR-19b

Rab8b mmu-miR-19b

Spry4 mmu-miR-19b

Stk35 mmu-miR-19b

Stk38 mmu-miR-19b

Tgfbr2 mmu-miR-19b

Tgif1 mmu-miR-19b

Tgm3 mmu-miR-19b

Tgoln1 mmu-miR-19b

Tmem1 mmu-miR-19b

Tmem151b mmu-miR-19b

Vamp3 mmu-miR-19b

Vapb mmu-miR-19b

Vps37a mmu-miR-19b

Vps37b mmu-miR-19b

Zdhhc18 mmu-miR-19b

Zdhhc7 mmu-miR-19b

Zer1 mmu-miR-19b

Zfand5 mmu-miR-19b

Gene Symbol miRNA

Zfp113 mmu-miR-19b

Zfp238 mmu-miR-19b

Acvr1c mmu-miR-181b

Acvr2a mmu-miR-181b

Adam11 mmu-miR-181b

Adam22 mmu-miR-181b

Atp2a2 mmu-miR-181b

Atp2b1 mmu-miR-181b

Bat2d mmu-miR-181b

Baz1a mmu-miR-181b

Baz2a mmu-miR-181b

Baz2b mmu-miR-181b

Bcl2 mmu-miR-181b

Bcl2l11 mmu-miR-181b

Bcl9 mmu-miR-181b

Bclaf1 mmu-miR-181b

Braf mmu-miR-181b

Brd1 mmu-miR-181b

Btbd14a mmu-miR-181b

Btbd3 mmu-miR-181b

Cacna2d2 mmu-miR-181b

Calm1 mmu-miR-181b

Camk2g mmu-miR-181b

Camsap1 mmu-miR-181b

Camsap1l1 mmu-miR-181b

Camta2 mmu-miR-181b

Card11 mmu-miR-181b

Carm1 mmu-miR-181b

Cbfa2t2 mmu-miR-181b

Cbfa2t3 mmu-miR-181b

Cblb mmu-miR-181b

Ccdc52 mmu-miR-181b

Ccnj mmu-miR-181b

Ccnk mmu-miR-181b

Cd163 mmu-miR-181b

Cd2ap mmu-miR-181b

Cd4 mmu-miR-181b

Cdk8 mmu-miR-181b

Cdkl2 mmu-miR-181b

Cdkn2aip mmu-miR-181b

Cdon mmu-miR-181b

Centb2 mmu-miR-181b

Chd9 mmu-miR-181b

Clasp1 mmu-miR-181b

Cnksr2 mmu-miR-181b

Gene Symbol miRNA

Cnksr3 mmu-miR-181b

Col16a1 mmu-miR-181b

Col5a1 mmu-miR-181b

Cops2 mmu-miR-181b

Cpd mmu-miR-181b

Cpne2 mmu-miR-181b

Creb1 mmu-miR-181b

Csnk1g3 mmu-miR-181b

Csnk2a2 mmu-miR-181b

Ctdspl mmu-miR-181b

Cttnbp2nl mmu-miR-181b

Cul3 mmu-miR-181b

Dazap2 mmu-miR-181b

Dcbld2 mmu-miR-181b

Dclk1 mmu-miR-181b

Ddit4 mmu-miR-181b

Ddx3x mmu-miR-181b

Dek mmu-miR-181b

Depdc6 mmu-miR-181b

Derl1 mmu-miR-181b

Dip2c mmu-miR-181b

Dlg2 mmu-miR-181b

Egr1 mmu-miR-181b

Eif4a2 mmu-miR-181b

Eln mmu-miR-181b

Elp4 mmu-miR-181b

En2 mmu-miR-181b

Entpd6 mmu-miR-181b

Etohi1 mmu-miR-181b

Ets1 mmu-miR-181b

Etv6 mmu-miR-181b

Evx1 mmu-miR-181b

Eya3 mmu-miR-181b

Fbxl3 mmu-miR-181b

Fbxo11 mmu-miR-181b

Fbxo33 mmu-miR-181b

Fbxo41 mmu-miR-181b

Fign mmu-miR-181b

Fkbp1a mmu-miR-181b

Flt1 mmu-miR-181b

Fmnl2 mmu-miR-181b

Fmr1 mmu-miR-181b

Gas7 mmu-miR-181b

Gata6 mmu-miR-181b

Gdi1 mmu-miR-181b

Gene Symbol miRNA

Gdpd1 mmu-miR-181b

Gfpt1 mmu-miR-181b

Gigyf1 mmu-miR-181b

Hoxa1 mmu-miR-181b

Hoxa11 mmu-miR-181b

Hrb mmu-miR-181b

Igf2bp2 mmu-miR-181b

Igf2bp3 mmu-miR-181b

Igsf11 mmu-miR-181b

Ipmk mmu-miR-181b

Ipo5 mmu-miR-181b

Ipo8 mmu-miR-181b

Ippk mmu-miR-181b

Iqsec2 mmu-miR-181b

Itga1 mmu-miR-181b

Itga3 mmu-miR-181b

Jarid2 mmu-miR-181b

Kcna4 mmu-miR-181b

Kcnc2 mmu-miR-181b

Kcnh1 mmu-miR-181b

Klf15 mmu-miR-181b

Kpna1 mmu-miR-181b

Kpnb1 mmu-miR-181b

L1cam mmu-miR-181b

Larp4 mmu-miR-181b

Lmx1a mmu-miR-181b

Lrba mmu-miR-181b

Lrig2 mmu-miR-181b

Lrp12 mmu-miR-181b

Lrp4 mmu-miR-181b

Lrrc32 mmu-miR-181b

Luzp1 mmu-miR-181b

Lycat mmu-miR-181b

Lyrm1 mmu-miR-181b

Maea mmu-miR-181b

Mamdc2 mmu-miR-181b

Man2a1 mmu-miR-181b

Map3k10 mmu-miR-181b

Map3k7ip3 mmu-miR-181b

Map4k4 mmu-miR-181b

Mapk1 mmu-miR-181b

Mapre2 mmu-miR-181b

Pcdha1 mmu-miR-181b

Pcdha10 mmu-miR-181b

Pcdha11 mmu-miR-181b

Gene Symbol miRNA

Pcdha12 mmu-miR-181b

Pcdha2 mmu-miR-181b

Pcdha3 mmu-miR-181b

Pcdha4 mmu-miR-181b

Pcdha5 mmu-miR-181b

Pcdha6 mmu-miR-181b

Pcdha7 mmu-miR-181b

Pcdha8 mmu-miR-181b

Pcdha9 mmu-miR-181b

Pcdhac1 mmu-miR-181b

Pcdhac2 mmu-miR-181b

Prtg mmu-miR-181b

Psap mmu-miR-181b

Psmf1 mmu-miR-181b

Ptbp2 mmu-miR-181b

Pten mmu-miR-181b

Ptpn9 mmu-miR-181b

Punc mmu-miR-181b

Qk mmu-miR-181b

Qser1 mmu-miR-181b

Rab11fip2 mmu-miR-181b

Rab6b mmu-miR-181b

Rab8b mmu-miR-181b

Rabgef1 mmu-miR-181b

Rad21 mmu-miR-181b

Rad23b mmu-miR-181b

Rai1 mmu-miR-181b

Runx1 mmu-miR-181b

S1pr1 mmu-miR-181b

Sall4 mmu-miR-181b

Scamp2 mmu-miR-181b

Schip1 mmu-miR-181b

Sec24c mmu-miR-181b

Sel1l mmu-miR-181b

Sema4g mmu-miR-181b

Senp1 mmu-miR-181b

Shoc2 mmu-miR-181b

Sim1 mmu-miR-181b

Sin3b mmu-miR-181b

Sipa1l2 mmu-miR-181b

Sirt1 mmu-miR-181b

Six2 mmu-miR-181b

Six4 mmu-miR-181b

Smad7 mmu-miR-181b

Snai2 mmu-miR-181b

Gene Symbol miRNA

Snn mmu-miR-181b

Sos1 mmu-miR-181b

Sox5 mmu-miR-181b

Sox6 mmu-miR-181b

Sp1 mmu-miR-181b

Spag9 mmu-miR-181b

Specc1l mmu-miR-181b

Spire1 mmu-miR-181b

Spry4 mmu-miR-181b

Srpk2 mmu-miR-181b

Ss18l1 mmu-miR-181b

Ssx2ip mmu-miR-181b

St8sia4 mmu-miR-181b

Stim2 mmu-miR-181b

Tbcel mmu-miR-181b

Tbl1x mmu-miR-181b

Tbl1xr1 mmu-miR-181b

Tbpl1 mmu-miR-181b

Tcerg1 mmu-miR-181b

Tgfbr1 mmu-miR-181b

Tgfbr3 mmu-miR-181b

Thoc2 mmu-miR-181b

Thrb mmu-miR-181b

Timp3 mmu-miR-181b

Tlk1 mmu-miR-181b

Tm9sf3 mmu-miR-181b

Tm9sf4 mmu-miR-181b

Tmed4 mmu-miR-181b

Ube2h mmu-miR-181b

Ube2l3 mmu-miR-181b

Ube2n mmu-miR-181b

Ube3c mmu-miR-181b

Ubp1 mmu-miR-181b

Unc5a mmu-miR-181b

Unc84a mmu-miR-181b

Unkl mmu-miR-181b

Usp12 mmu-miR-181b

Usp33 mmu-miR-181b

Usp42 mmu-miR-181b

Vsx1 mmu-miR-181b

Wdr82 mmu-miR-181b

Whdc1 mmu-miR-181b

Wnk1 mmu-miR-181b

Wnt9a mmu-miR-181b

Wwc2 mmu-miR-181b

Gene Symbol miRNA

Xiap mmu-miR-181b

Xk mmu-miR-181b

Xpo7 mmu-miR-181b

Yod1 mmu-miR-181b

Ywhag mmu-miR-181b

Yy1 mmu-miR-181b

Zadh2 mmu-miR-181b

Zbtb34 mmu-miR-181b

Zbtb4 mmu-miR-181b

A2bp1 mmu-miR-328

Add2 mmu-miR-328

Arpp19 mmu-miR-328

Barhl1 mmu-miR-328

Cenpo mmu-miR-328

Cnot2 mmu-miR-328

Crk mmu-miR-328

Ebf3 mmu-miR-328

Edaradd mmu-miR-328

Esco1 mmu-miR-328

Etv6 mmu-miR-328

Fam199x mmu-miR-328

Fgf16 mmu-miR-328

Fzd7 mmu-miR-328

Gm2a mmu-miR-328

Gprc5c mmu-miR-328

Gtdc1 mmu-miR-328

H13 mmu-miR-328

H2afx mmu-miR-328

Heyl mmu-miR-328

Hist1h4i mmu-miR-328

Hist2h4 mmu-miR-328

Hpcal4 mmu-miR-328

Igf1r mmu-miR-328

Ipmk mmu-miR-328

Itga5 mmu-miR-328

Klhdc5 mmu-miR-328

Lgr4 mmu-miR-328

Lmod1 mmu-miR-328

Lnp mmu-miR-328

Man1c1 mmu-miR-328

Mbnl3 mmu-miR-328

Mecp2 mmu-miR-328

Myst4 mmu-miR-328

Ndor1 mmu-miR-328

Nexn mmu-miR-328

Gene Symbol miRNA

Nr3c1 mmu-miR-328

Pde4dip mmu-miR-328

Pef1 mmu-miR-328

Pim1 mmu-miR-328

Prdm16 mmu-miR-328

Psme3 mmu-miR-328

Pten mmu-miR-328

Ptpn9 mmu-miR-328

Ptprf mmu-miR-328

Rab3c mmu-miR-328

Rad23b mmu-miR-328

Rbm35a mmu-miR-328

Rs1 mmu-miR-328

Rsbn1l mmu-miR-328

Rwdd2b mmu-miR-328

Slc17a2 mmu-miR-328

Slc27a4 mmu-miR-328

Slc7a7 mmu-miR-328

Snrk mmu-miR-328

Sox11 mmu-miR-328

Stxbp5l mmu-miR-328

Tcf7l2 mmu-miR-328

Tesk2 mmu-miR-328

Tle1 mmu-miR-328

Tox4 mmu-miR-328

Ttc38 mmu-miR-328

Ube2z mmu-miR-328

Ubfd1 mmu-miR-328

Usp37 mmu-miR-328

Zbtb4 mmu-miR-328

Zfp131 mmu-miR-328

Zfp46 mmu-miR-328

Aass mmu-miR-335-3p

Abhd5 mmu-miR-335-3p

Acp1 mmu-miR-335-3p

Acvr1c mmu-miR-335-3p

Adam10 mmu-miR-335-3p

Adamts17 mmu-miR-335-3p

Adamts8 mmu-miR-335-3p

Aebp2 mmu-miR-335-3p

Ahdc1 mmu-miR-335-3p

Akap2 mmu-miR-335-3p

Als2cr13 mmu-miR-335-3p

Als2cr4 mmu-miR-335-3p

Anapc11 mmu-miR-335-3p

Gene Symbol miRNA

Ank1 mmu-miR-335-3p

Ank3 mmu-miR-335-3p

Arfgef2 mmu-miR-335-3p

Arhgef9 mmu-miR-335-3p

Arl15 mmu-miR-335-3p

Armc2 mmu-miR-335-3p

Atad5 mmu-miR-335-3p

Atp1a2 mmu-miR-335-3p

Atp1b4 mmu-miR-335-3p

Atp2a2 mmu-miR-335-3p

Atp6v1b2 mmu-miR-335-3p

Atp6v1g1 mmu-miR-335-3p

B3gnt9-ps mmu-miR-335-3p

B4galt4 mmu-miR-335-3p

Barhl2 mmu-miR-335-3p

Bcl11b mmu-miR-335-3p

Bdnf mmu-miR-335-3p

Brd1 mmu-miR-335-3p

Btf3l4 mmu-miR-335-3p

C1galt1 mmu-miR-335-3p

Cacnb4 mmu-miR-335-3p

Camk2d mmu-miR-335-3p

Cap2 mmu-miR-335-3p

Cbfa2t3 mmu-miR-335-3p

Cbx3 mmu-miR-335-3p

Cbx5 mmu-miR-335-3p

Ccdc38 mmu-miR-335-3p

Ccnc mmu-miR-335-3p

Cd2ap mmu-miR-335-3p

Cdc37l1 mmu-miR-335-3p

Cdc73 mmu-miR-335-3p

Cdcp1 mmu-miR-335-3p

Cds1 mmu-miR-335-3p

Cep110 mmu-miR-335-3p

Chd2 mmu-miR-335-3p

Cited2 mmu-miR-335-3p

Clcn4-2 mmu-miR-335-3p

Cnot2 mmu-miR-335-3p

Cntln mmu-miR-335-3p

Col17a1 mmu-miR-335-3p

Col4a1 mmu-miR-335-3p

Col8a1 mmu-miR-335-3p

Cplx3 mmu-miR-335-3p

Crispld2 mmu-miR-335-3p

Csnk1e mmu-miR-335-3p

Gene Symbol miRNA

Ctla4 mmu-miR-335-3p

Ctnnbip1 mmu-miR-335-3p

Cul3 mmu-miR-335-3p

Cyfip2 mmu-miR-335-3p

Cyld mmu-miR-335-3p

Cyp7b1 mmu-miR-335-3p

Cysltr1 mmu-miR-335-3p

Dab1 mmu-miR-335-3p

Dach1 mmu-miR-335-3p

Dnaja4 mmu-miR-335-3p

Dnajb5 mmu-miR-335-3p

Dscaml1 mmu-miR-335-3p

Dusp22 mmu-miR-335-3p

Dyrk1a mmu-miR-335-3p

Eaf1 mmu-miR-335-3p

Edem3 mmu-miR-335-3p

Egln1 mmu-miR-335-3p

Eif2s3x mmu-miR-335-3p

Eif4b mmu-miR-335-3p

Elmo2 mmu-miR-335-3p

Eml4 mmu-miR-335-3p

Eml5 mmu-miR-335-3p

Ep300 mmu-miR-335-3p

Epc1 mmu-miR-335-3p

Epha5 mmu-miR-335-3p

Erc2 mmu-miR-335-3p

Ermn mmu-miR-335-3p

Esyt2 mmu-miR-335-3p

Etv3 mmu-miR-335-3p

Evi2b mmu-miR-335-3p

Eya3 mmu-miR-335-3p

Eya4 mmu-miR-335-3p

F2r mmu-miR-335-3p

Fa2h mmu-miR-335-3p

Fam126b mmu-miR-335-3p

Fam49a mmu-miR-335-3p

Fam81a mmu-miR-335-3p

Fat3 mmu-miR-335-3p

Fbxo42 mmu-miR-335-3p

Flrt3 mmu-miR-335-3p

Flywch1 mmu-miR-335-3p

Fmn2 mmu-miR-335-3p

Fndc5 mmu-miR-335-3p

Fos mmu-miR-335-3p

Frap1 mmu-miR-335-3p

Gene Symbol miRNA

Fubp1 mmu-miR-335-3p

Fzd5 mmu-miR-335-3p

Gabrb3 mmu-miR-335-3p

Gad2 mmu-miR-335-3p

Galnt7 mmu-miR-335-3p

Glcci1 mmu-miR-335-3p

Gli3 mmu-miR-335-3p

Gosr1 mmu-miR-335-3p

Gpr180 mmu-miR-335-3p

Gpr82 mmu-miR-335-3p

Grik2 mmu-miR-335-3p

Gspt1 mmu-miR-335-3p

Gys1 mmu-miR-335-3p

Hcrtr2 mmu-miR-335-3p

Hhip mmu-miR-335-3p

Hist1h2ac mmu-miR-335-3p

Hlcs mmu-miR-335-3p

Hlf mmu-miR-335-3p

Hmgn2 mmu-miR-335-3p

Hnrnpa3 mmu-miR-335-3p

Homer1 mmu-miR-335-3p

Hook3 mmu-miR-335-3p

Htr7 mmu-miR-335-3p

Igf1r mmu-miR-335-3p

Igf2bp1 mmu-miR-335-3p

Igsf3 mmu-miR-335-3p

Inhbb mmu-miR-335-3p

Ippk mmu-miR-335-3p

Itga6 mmu-miR-335-3p

Itga8 mmu-miR-335-3p

Itgb1bp1 mmu-miR-335-3p

Itgb6 mmu-miR-335-3p

Jag1 mmu-miR-335-3p

Jmy mmu-miR-335-3p

Kcnc2 mmu-miR-335-3p

Kctd9 mmu-miR-335-3p

Kitl mmu-miR-335-3p

Klf12 mmu-miR-335-3p

Kpna4 mmu-miR-335-3p

Kras mmu-miR-335-3p

Leng1 mmu-miR-335-3p

Lingo1 mmu-miR-335-3p

Lmo4 mmu-miR-335-3p

Lnx2 mmu-miR-335-3p

Lphn2 mmu-miR-335-3p

Gene Symbol miRNA

Lrp4 mmu-miR-335-3p

Lrrc40 mmu-miR-335-3p

Lrrtm3 mmu-miR-335-3p

Luc7l3 mmu-miR-335-3p

Mafb mmu-miR-335-3p

Map3k12 mmu-miR-335-3p

Mapk6 mmu-miR-335-3p

Mbnl3 mmu-miR-335-3p

Mbtd1 mmu-miR-335-3p

Mctp2 mmu-miR-335-3p

Med1 mmu-miR-335-3p

Med13 mmu-miR-335-3p

Med14 mmu-miR-335-3p

Med22 mmu-miR-335-3p

Mex3a mmu-miR-335-3p

Mga mmu-miR-335-3p

Mlec mmu-miR-335-3p

Mtmr10 mmu-miR-335-3p

Mtmr6 mmu-miR-335-3p

Mycn mmu-miR-335-3p

Napg mmu-miR-335-3p

Narg1 mmu-miR-335-3p

Nars2 mmu-miR-335-3p

Ncam1 mmu-miR-335-3p

Ncoa2 mmu-miR-335-3p

Ncoa5 mmu-miR-335-3p

Ncoa7 mmu-miR-335-3p

Ndst3 mmu-miR-335-3p

Nedd4 mmu-miR-335-3p

Neurog1 mmu-miR-335-3p

Nfia mmu-miR-335-3p

Nit1 mmu-miR-335-3p

Nkx2-2 mmu-miR-335-3p

Npas3 mmu-miR-335-3p

Nr1h5 mmu-miR-335-3p

Nr3c2 mmu-miR-335-3p

Nrarp mmu-miR-335-3p

Nrxn1 mmu-miR-335-3p

Nrxn3 mmu-miR-335-3p

Nudt15 mmu-miR-335-3p

Ogn mmu-miR-335-3p

Ogt mmu-miR-335-3p

Olfm3 mmu-miR-335-3p

Onecut2 mmu-miR-335-3p

Osbp2 mmu-miR-335-3p

Gene Symbol miRNA

Pabpc4l mmu-miR-335-3p

Paip1 mmu-miR-335-3p

Pappa mmu-miR-335-3p

Parva mmu-miR-335-3p

Pax8 mmu-miR-335-3p

Pcdh15 mmu-miR-335-3p

Pcnx mmu-miR-335-3p

Pdpk1 mmu-miR-335-3p

Pds5b mmu-miR-335-3p

Pdzd2 mmu-miR-335-3p

Pdzd7 mmu-miR-335-3p

Phf2 mmu-miR-335-3p

Phf8 mmu-miR-335-3p

Pitpnm2 mmu-miR-335-3p

Pitx2 mmu-miR-335-3p

Pkib mmu-miR-335-3p

Plekhf2 mmu-miR-335-3p

Plekhh2 mmu-miR-335-3p

Plod2 mmu-miR-335-3p

Plxna2 mmu-miR-335-3p

Pou3f3 mmu-miR-335-3p

Ppig mmu-miR-335-3p

Ppil1 mmu-miR-335-3p

Ppm1e mmu-miR-335-3p

Ppp1r12a mmu-miR-335-3p

Prkcz mmu-miR-335-3p

Prrx1 mmu-miR-335-3p

Ptprd mmu-miR-335-3p

Rab1 mmu-miR-335-3p

Rab38 mmu-miR-335-3p

Rab39b mmu-miR-335-3p

Rag1 mmu-miR-335-3p

Rap1b mmu-miR-335-3p

Rap2b mmu-miR-335-3p

Rasgef1b mmu-miR-335-3p

Rbl2 mmu-miR-335-3p

Rbm28 mmu-miR-335-3p

Rc3h1 mmu-miR-335-3p

Rcbtb1 mmu-miR-335-3p

Rgs7bp mmu-miR-335-3p

Rnf13 mmu-miR-335-3p

Rnf145 mmu-miR-335-3p

Rnf2 mmu-miR-335-3p

Rnft1 mmu-miR-335-3p

Rp2h mmu-miR-335-3p

Gene Symbol miRNA

Rpe mmu-miR-335-3p

Rpgrip1l mmu-miR-335-3p

Rps6kc1 mmu-miR-335-3p

Rsrc1 mmu-miR-335-3p

Runx1t1 mmu-miR-335-3p

Ryk mmu-miR-335-3p

Samd5 mmu-miR-335-3p

Sbno1 mmu-miR-335-3p

Scfd2 mmu-miR-335-3p

Scg5 mmu-miR-335-3p

Sema3c mmu-miR-335-3p

Setd7 mmu-miR-335-3p

Sfrs1 mmu-miR-335-3p

Sfrs3 mmu-miR-335-3p

Shisa6 mmu-miR-335-3p

Shroom3 mmu-miR-335-3p

Skil mmu-miR-335-3p

Smarce1 mmu-miR-335-3p

Sox11 mmu-miR-335-3p

Sox12 mmu-miR-335-3p

Sparc mmu-miR-335-3p

Spry2 mmu-miR-335-3p

Srpk2 mmu-miR-335-3p

Ssbp2 mmu-miR-335-3p

Stox2 mmu-miR-335-3p

Strap mmu-miR-335-3p

Stxbp5l mmu-miR-335-3p

Susd5 mmu-miR-335-3p

Syncrip mmu-miR-335-3p

Syt11 mmu-miR-335-3p

Syt4 mmu-miR-335-3p

Syt7 mmu-miR-335-3p

Tanc1 mmu-miR-335-3p

Taok1 mmu-miR-335-3p

Tbc1d9 mmu-miR-335-3p

Tbk1 mmu-miR-335-3p

Tcf4 mmu-miR-335-3p

Tead1 mmu-miR-335-3p

Tm7sf3 mmu-miR-335-3p

Tm9sf3 mmu-miR-335-3p

Tmed4 mmu-miR-335-3p

Tmeff2 mmu-miR-335-3p

Tmem106b mmu-miR-335-3p

Tmem33 mmu-miR-335-3p

Tmem69 mmu-miR-335-3p

Gene Symbol miRNA

Tmtc3 mmu-miR-335-3p

Tnrc18 mmu-miR-335-3p

Tnrc6b mmu-miR-335-3p

Toe1 mmu-miR-335-3p

Tox3 mmu-miR-335-3p

Trove2 mmu-miR-335-3p

Trpc3 mmu-miR-335-3p

Trps1 mmu-miR-335-3p

Tshz1 mmu-miR-335-3p

Tspan1 mmu-miR-335-3p

Ttc21b mmu-miR-335-3p

Txlng mmu-miR-335-3p

Ube2q1 mmu-miR-335-3p

Ubn2 mmu-miR-335-3p

Ubxd8 mmu-miR-335-3p

Usp45 mmu-miR-335-3p

Vdac2 mmu-miR-335-3p

Vsig10 mmu-miR-335-3p

Wdr26 mmu-miR-335-3p

Xiap mmu-miR-335-3p

Yaf2 mmu-miR-335-3p

Zbtb7a mmu-miR-335-3p

Zc3h14 mmu-miR-335-3p

Zcchc2 mmu-miR-335-3p

Zcchc24 mmu-miR-335-3p

Zfp238 mmu-miR-335-3p

Zfp36l1 mmu-miR-335-3p

Zfp36l2 mmu-miR-335-3p

Zfp449 mmu-miR-335-3p

Zfp606 mmu-miR-335-3p

Zfp655 mmu-miR-335-3p

Acvr1c mmu-miR-468

Adamts6 mmu-miR-468

Appl1 mmu-miR-468

Arhgap26 mmu-miR-468

Asxl2 mmu-miR-468

Bdnf mmu-miR-468

Cask mmu-miR-468

Cisd2 mmu-miR-468

Cpeb3 mmu-miR-468

Csn2 mmu-miR-468

Dab2 mmu-miR-468

Ddx3x mmu-miR-468

Dmd mmu-miR-468

Dmxl1 mmu-miR-468

Gene Symbol miRNA

Dnalc1 mmu-miR-468

Foxp2 mmu-miR-468

Gpr22 mmu-miR-468

Hnrnpa2b1 mmu-miR-468

Itga9 mmu-miR-468

Kcna1 mmu-miR-468

Kitl mmu-miR-468

Marcks mmu-miR-468

Mbnl1 mmu-miR-468

Mtap1b mmu-miR-468

Nbea mmu-miR-468

Ndst1 mmu-miR-468

Negr1 mmu-miR-468

Nlk mmu-miR-468

Npal2 mmu-miR-468

Ntng1 mmu-miR-468

Olfm3 mmu-miR-468

Opcml mmu-miR-468

Gene Symbol miRNA

Otud7a mmu-miR-468

Paqr8 mmu-miR-468

Pdcd7 mmu-miR-468

Pdzd2 mmu-miR-468

Ptger2 mmu-miR-468

Ptp4a1 mmu-miR-468

Ptprj mmu-miR-468

Rai1 mmu-miR-468

Rtf1 mmu-miR-468

Rtkn2 mmu-miR-468

Spire1 mmu-miR-468

Tmem1 mmu-miR-468

Tox mmu-miR-468

Ube2d2 mmu-miR-468

Uqcc mmu-miR-468

Vipr1 mmu-miR-468

Zfp608 mmu-miR-468

MiRNAs are identified from microarray analysis (Table S1).
