# Supplementary material for: Androgen-Responsive MicroRNAs in Mouse Sertoli Cells
Source: PLoS One. 2012 Jul 20;7(7):e41146. doi: 10.1371/journal.pone.0041146 (PMC3401116; doi:10.1371/journal.pone.0041146)
Supplement: Table S3 — Primers used in this study. (DOC) [file pone.0041146.s007.doc]

**Table S3. Primers used in this study.**

RNU19 F TGT GGT GCC CGA GAT CGT

RNU19 R TGG GAG CCG ACC CTT AGT AA

5S rRNA F AGG GTC GGG CCT GGT TAG TA

5S rRNA R CCT ACA GCA CCC GGT ATT CC

miR-471 TAC GTA GTA TAG TGC TTT TCA

miR-741 TGA GAG ATG CCA TTC TAT GTA

miR-201 TAC TCA GTA AGG CAT TGT TCT T

miR-463 TGA TAG ACA CCA TAT AAG GTA G

miR-743a GAA AGA CAC CAA GCT GAG

miR-15b CAC ATC ATG GTT TAC A

miR-203 ATG TTT AGG ACC ACT AG

miR-25 CTT GTC TCG GTC TGA

miR-34c TAA CCA CAC AGC CAG G

miR-375 TTT GTT CGT TCG GCT CGC GTG A

miR-449a TGG CAG TGT ATT GTT

miR-547 CTT GGT ACA TCT TTG AGT GAG

miR-741 GAT GCC ATT CTA TG

miR-871-3p TGA CTG GCA CCA TTC TGG ATA AT

miR-878-5p TAT CTA GTT GGA TGT CAA GAC A

miR-880 TAC TCC ATC CTC TCT GA

miR-328 CTG GCC CTC TCT GCC CTT

miR-18a* CCT AAG TGC TCC TTC TG

miR-335-3p TTT CAT TAT TGC TCC TGA CC

miR-468 TAT GAC TGA TGT GCG TGT G

Foxd1 F CGA GAT CTG CGA GTT CAT CA

Foxd1 R TTG ACG AAG CAG TCG TTG AG

Dsc1 F GGT CAA GGA ATC AAA ACA CAG C

Dsc1 R CCA AGC CGA GGT TGA GTG AAA
